# GABAergic signaling contributes to tumor cell invasion and poor overall survival in colorectal cancer

**DOI:** 10.1038/s41388-025-03546-2

**Published:** 2025-08-24

**Authors:** Carly Strelez, Francesca Battaglin, Rachel Perez, Yan Yang, Christopher Cherry, Joshua Millstein, Ah Young Yoon, John S. Chlystek, Ethan Canfield, Bethany Haliday, Curran Shah, Kimya Ghaffarian, Shivani Soni, Hannah Jiang, Roy Lau, Aaron Schatz, Yuyuan Zhou, Daniel Mulkerin, Fang-Shu Ou, Alan P. Venook, Federico Innocenti, Josh Neman, Jonathan E. Katz, Heinz-Josef Lenz, Shannon M. Mumenthaler

**Affiliations:** 1 Ellison Medical Institute, Los Angeles, CA USA; 2 Division of Oncology, Norris Comprehensive Cancer Center, Keck School of Medicine, University of Southern California, Los Angeles, CA USA; 3 Department of Population and Public Health Sciences, Keck School of Medicine, University of Southern California, Los Angeles, CA USA; 4 Department of Biomedical Engineering, Viterbi School of Engineering, University of Southern California, Los Angeles, CA USA; 5 Department of Chemical Engineering, Viterbi School of Engineering, University of Southern California, Los Angeles, CA USA; 6 University of Rochester Wilmot Cancer Institute, Rochester, NY USA; 7 Alliance Statistics and Data Management Center, Mayo Clinic, Rochester, MN USA; 8 University of California, San Francisco, San Francisco, CA USA; 9 University of North Carolina at Chapel Hill, Chapel Hill, NC USA; 10 Department of Neurological Surgery, University of Southern California, Los Angeles, CA USA; 11 Keck School of Medicine, University of Southern California, Los Angeles, CA USA

**Keywords:** Colorectal cancer, Cancer models, Cancer microenvironment

## Abstract

Alterations in neurotransmitter signaling can influence colorectal cancer (CRC). In a large, randomized Phase III clinical trial (CALGB/SWOG 80405) involving patients with metastatic CRC, high expression of gamma-aminobutyric acid (GABA) pathway gene *GAD1* and low expression of *ABAT*, indicative of a GABAergic environment, were associated with worse progression-free survival and overall survival outcomes. A metastasis map of human cancer cell lines (MetMap) and functional studies using a microfluidic tumor-on-chip platform demonstrated that high *GAD1* expression correlates with increased metastatic potential. Knockdown and pharmacological inhibition of *GAD1* reduced tumor invasion, while exogenous GABA promoted invasion. Tumor-derived GABA was elevated in Ras-altered tumors. Furthermore, analysis of publicly available data confirmed that higher GAD1 expression is associated with worse outcomes in Ras-mutant tumors. These findings establish a role for GABA signaling in tumor invasiveness, particularly in Ras-altered CRC. This study demonstrates using clinical data to inform new discoveries and highlights the need for advanced preclinical model systems that more accurately reflect human physiology to explore these findings.

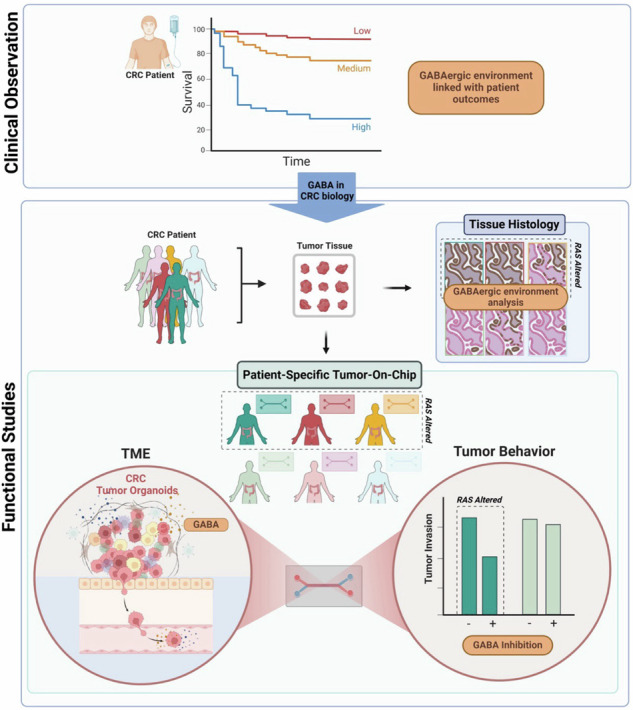

## Introduction

Cancer, a complex and multifaceted disease, continues to challenge our understanding and treatment efforts. While remarkable strides have been made in elucidating the genetic underpinnings of various malignancies [1, 2], there remains a critical need to comprehensively explore the tumor microenvironment (TME) and its intricate interplay with cancer progression. Recent studies suggest that alterations in neurotransmitter signaling within the gut may contribute to the initiation and progression of colorectal cancer (CRC) [3, 4]. Dysregulation of serotonin, for instance, has been linked to changes in gut motility and inflammation, which can create an environment conducive to CRC development [5, 6]. Additionally, gamma-aminobutyric acid (GABA) levels and GAD1 activity (glutamate decarboxylase 1, the enzyme responsible for catalyzing the production of GABA from L-glutamic acid) have been shown to be elevated in colon cancer tissues as compared to normal colon tissues [7, 8]. Alterations in GABA signaling, including GABA receptor activation and GABA production via GAD1, have been found to affect both the tumor and TME in CRC [7, 9–12]. However, in vitro data has been inconsistent, and evaluating the relevance of these findings to humans has been challenging due to the limitations in current model systems. To establish a link between GABAergic signaling and patient outcomes, we combined clinical trial data with mechanistic insights gained from a microphysiological system (MPS) recapitulating aspects of the biophysical and biochemical TME in CRC.

MPS’s represent a paradigm shift in our ability to model the intricacies of human (patho)physiology in vitro [13]. Microfluidic organ-on-chip (OOC) technology emerges as one such promising MPS solution, offering a more comprehensive platform for investigating normal organ development and disease pathologies, including cancer, by integrating vascular networks [14–16] and simulating biochemical and biophysical conditions [17, 18]. Specifically, tumor-on-chip models have been invaluable for exploring the complexities of the TME and its interactions, driving advancements in tumor modeling and metastasis, drug screening, personalized medicine, and immunotherapy approaches [19–23]. More recently, combining OOC platforms with patient-derived organoids enables a more accurate replication of individual tumor characteristics and heterogeneity [24–27].

In this manuscript, we developed a CRC organoid-on-chip platform that mimics the epithelial-endothelial interface and incorporates key mechanical forces found in the gut, such as fluid flow and peristalsis-like motions. This system enabled the investigation of GABA-related patient heterogeneity, tumor microenvironment interactions, and cancer invasion. We demonstrated that tumor-derived GABA production, mediated by glutamate decarboxylase 1 (*GAD1*), is associated with an invasive phenotype in Ras-altered colon cancers. These findings suggest that the inhibition of GABAergic signaling may be a viable strategy in Ras-altered cancers, which is supported by clinical data as well as functional studies involving genetic and pharmacological inhibition of *GAD1* in cell lines and patient-derived models.

## Materials and Methods

### Ethics Statement

For the OOC studies, the use of patient samples was reviewed and approved by the USC Biomedical Institutional Review Board Committee, under protocol number HS-06–00678. All patients provided written informed consent prior to sample collection. Samples were de-identified to research staff. For the patient outcome analysis, all patients provided informed consent to make their data publicly available. All methods were performed in accordance with the relevant guidelines and regulations

### Patient Outcome Analysis in the CALGB/SWOG 80405 Trial

The association between GABA pathway genes expression and clinical outcomes in patients receiving targeted treatment was assessed in 433 patients with available RNA-sequencing (RNA-seq, Illumina HiSeq 2500) data from the phase III Cancer and Leukemia Group B (CALGB, now part of the Alliance for Clinical Trials in Oncology)/SWOG 80405 trial comparing first-line cetuximab and bevacizumab, in combination with either FOLFOX or FOLFIRI, in patients with mCRC.

We evaluated the association of the tumor tissue expression of 25 genes in the GABA pathway (*GABBR1*, *GABBR2*, *GABRA1*, *GABRA2*, *GABRA4*, *GABRA5*, *GABRA6*, *GABRB1*, *GABRB3*, *GABRD*, *GABRE*, *GABRG1*, *GABRG2*, *GABRG3*, *GABRQ*, *GABRR1*, *GABRR2*, *ABAT*, *ALDH5A1*, *GAD1*, *GAD2*, *SLC6A1*, *SLC6A13*, *SLC6A11*, *SLC6A12*) with survival. OS was defined as the time from the date of randomization to death from any cause. PFS was defined as the time from randomization to disease progression or death from any cause, whichever was earlier. Patients who were alive were censored for OS at the last follow-up. Patients who were alive and progression-free were censored for PFS at the last disease assessment. The Kaplan-Meier method was used to estimate the distribution of OS and PFS between groups. The associations between categorical gene expression (categorized into tertiles [T1 low, T2 middle, and T3 high]) and outcomes were assessed via the log-rank test. The associations between continuous gene expression (per standard deviation increase) and outcomes were assessed using a Cox proportional hazards model with a likelihood ratio test. Variables adjusted in the models are age, sex, ECOG performance status, tumor location, number of metastatic sites, *KRAS*, MSI status, treatment, chemotherapy, and the first three principal components of the gene expression data, included to account for large-scale trends. HR and 95% CI were computed. Statistical significance was set at the 0.05 alpha level, and false discovery rate (FDR) was controlled with the Benjamini-Hochberg method. *GAD1* and *ABAT* expression were the genes that were consistently significant across treatment arms (see Table S2 for full results).

### Cell Lines

#### Commercially available cell lines

Human umbilical vein endothelial cells (HUVEC) expressing Red Fluorescent Protein (RFP) (Angio-Proteomie, #cAP-0001RFP) were expanded in EBM-2 media with EGM-2 SingleQuots Supplements (2% FBS, 1% Penicillin-Streptomycin (Pen-Strep), Hydrocortisone, hFGF-B, VEGF, R3-IGF-1, Ascorbic Acid, hEGF, and heparin in proprietary concentrations) (Lonza #CC-3162; supplemented with 1% Pen-Strep in lieu of Gentamicin). Human large intestine microvascular endothelial cells (HIMEC) (Cell Systems, #ACBRI 666) were labeled with LentiBrite^TM^-RFP (Millipore Sigma, #17-10409) and grown in Endothelial Cell Growth Medium MV2 (Promo Cell, #C-22121), with MV2 supplement pack (PromoCell, #C39221; supplemented with heat inactivated 5% FBS (Gemini, #100-500) in lieu of FCS) containing 5 ng/mL recombinant human epidermal growth factor (rhEGF), 10 ng/mL recombinant human basic fibroblast growth factor (rhbFGF), 20 ng/mL long R3 insulin-like growth factor (R3-IGF), 0.5 ng/mL recombinant human vascular endothelial growth factor 165 (rhVEGF), 1 μg/mL ascorbic acid, 0.2 μg/mL hydrocortisone and 50 μg mL-1 Primocin (InvivoGen #ant-pm-1). HIMEC cells used for chip conditions were between passage 6 and 8. Caco2 C2BBe1 cells (ATCC, #CRL-2102) were grown in DMEM (Gibco, #10569-010) with 10% fetal bovine serum (FBS) and 1% Pen-Strep. HCT116 (ATCC #CCL-247) were grown in McCoy’s 5 A media (Gibco, #16600-082) with 10% FBS and 1% Pen-Strep, labeled with LentiBrite Histone-H2B-GFP Lentiviral Biosensor (Millipore, #17-10229), and sorted to achieve a pure fluorescent population. *GAD1* human shRNA lentiviral particles (Santa Cruz Biotechnology, Inc., #sc- sc-35435-V) and control shRNA lentiviral particles (Santa Cruz Biotechnology, Inc., #sc-108080) were used to produce HCT116 H2B-GFP *GAD1* knock down cells at a multiplicity of infection (MOI) of 2 and 2 mg/mL of puromycin (Santa Cruz Biotechnology, #sc-108071) was used to select for knocked down cells. DLD1 cells were obtained from the Vogelstein Lab at Johns Hopkins University, labeled with MISSION TurboGFP Control Transduction particles (Millipore Sigma, #SHC003V), and 2 mg/mL of puromycin (Santa Cruz Biotechnology, #sc-108071) was used to select for knocked down cells. Cells were maintained in McCoy’s 5A media (Gibco, #16600-082) with 10% FBS and 1% Pen-Strep supplemented with 1 mg/mL of puromycin. All cells were cultured under standard laboratory conditions (5% CO_2_, 37 °C) and routinely tested (approximately every 6 months) for mycoplasma contamination in-house and STR sequencing at the Arizona Genetics Core.

#### Patient-derived samples

Tissue resections were received from the USC Norris Comprehensive Cancer Center following Institutional Review Board (IRB) approval (Protocol HS-06-00678; continuous approval date 08-02-2019) and patient consent. Tumor profiles, including known tumor mutations, sex, and treatment information, are detailed in Fig. 3A and Table S3. Human organoids were derived from CRC tumors via a previously described method [28, 29]. Briefly, tumor pieces were minced and enzymatically digested using 1.5 mg/mL collagenase (Millipore, #234155), 10 μM LY27632 (Millipore, #5.09228.0001), and 20 μg/mL hyaluronidase (MP Biomedicals, #0210074080) for 30 minutes at 37 ^o^C. Established organoid cell lines were expanded by plating organoids with basement membrane extract (BME; Cultrex, #3533-005-02) cultured in colon media (Advance DMEM F12 (Gibco, #12634-010), supplemented with 10% FBS (Gemini, 100-500), 1% Pen-Strep (Gemini 400-109), 100 ng/mL Noggin (Tonbo, #21-7075-U500), 50 ng/mL epidermal growth factor (EGF) (Life Technologies, #PHG0313), 10 mM SB202190 (Millipore Sigma, #S7067), 500 nM TGF-b RI Kinase Inhibitor IV (A83-01) (Millipore Sigma, #616454-2MG), 10 mM Nicotinamide (Millipore Sigma, #N0636), 1 x B27 (Gibco, #17504-001), 1mM N-acetylcysteine (Millipore Sigma, #A9165), 1 x N2 (Gibco, #17502-048), 1 x HEPES (Gibco, #15630-080), 1 x GlutaMax (Gibco, #35050-061) at 5% CO_2_, 37°C. Media was replaced every 2-3 days. CRC organoids used for chip conditions were between passages 12 and 23. After establishment and expansion of organoids, ORG000US, ORG000UP, ORG000UK, ORG000U0, ORG000V8 (US, UP, UK, U0, and V8) were subsequently labeled with H2B-GFP lentivirus as previously described [30]. Briefly, organoids were dissociated with 50% TrypLE (Gibco; 12605-028) supplemented with 10 μM LY-27632 (Millipore, #5.09228.0001) and incubated at 37 °C for 5 min. After spinning, the pellet was resuspended in colon organoid media containing 5 µg mL-1 polybrene (Millipore Sigma; TR-1003-G) with LentiBrite Lentivirus H2B-GFP (Millipore Sigma; 1710229, 40 multiplicity of infection [MOI]) at 37 °C for 60 min. The pellet was then resuspended in BME and cultured and expanded. GFP-positive organoids were sorted with FACS using the ARIA IIu (BD Biosciences, San Diego, CA) or FACSMelody (BD Biosciences, San Diego, CA) to isolate only GFP positive-labeled cells.

### Colorectal Cancer Organ-on-Chip

Chips were acquired from Emulate, Inc and fabrication methods have been described previously by Emulate, Inc. In brief, the chips are made of transparent elastomeric polymer (polydimethylsiloxane, PDMS). They are divided into upper (1 mm high x 1 mm wide) and lower (0.2 mm high x 1 mm wide) microfluidic compartments separated by a thin, porous membrane (50 µm thick with 7 µm diameter pores; 17.1 mm^2^ co-culture region), with cancer cells in the upper compartment and endothelial cells in the lower compartment. Each compartment is coated with a tissue-specific ECM prior to cell seeding. The chips are attached to a Pod^TM^ portable module that encloses the chips to control sterility, holds inlet cell culture media and effluent, allows for monitoring via microscopy, and is designed to ensure no pressure differentials between channels. The chips and pod are then housed in an automated culture module instrument (Zoë^TM^ culture module and Orb^TM^ hub module, Emulate, Inc.) that controls the fluid flow and stretching forces while inside an incubator.

#### Caco2 + CRC Cell Lines

A CRC on-chip using commonly available CRC tumor cell lines has been previously described [31, 32]. Briefly, Emulate Chip-S1 Stretchable chips were activated by Emulate Reagent 1 and 2 (Emulate, Inc., ER-1 and ER-2) under UV light for 20 min at room temp. The epithelial and endothelial channels were coated with a mixture of 30 μg/mL type I collagen (Corning, #354249) and 100 μg/mL Matrigel (Corning, #356231) overnight at 4 ^o^C before washing with PBS. HUVEC cells (RFP-labeled or unlabeled) were seeded into the bottom channel (1–1.2 × 10^5^ cells in 20 μL; 5.8–7 × 10^5^ cells/cm^2^). The chips were inverted and incubated at 37 ^o^C for 2 h. After HUVEC attachment, Caco2 C2BBe1 cells were seeded into the top channel (50,000-62,500 cells in 50 μL; 3-3.7 × 10^5^ cells/cm^2^), incubated for 2 h at 37 ^o^C, and connected to flow. The chips were perfused with DMEM, 10% FBS, 1% Pen-Strep in the top channel and endothelial media (EBM-2 fully supplemented with 2% FBS, 1% Pen-Strep, Hydrocortisone, hFGF-B, VEGF, R3-IGF-1, Ascorbic Acid, hEGF, and heparin in proprietary concentrations) in the bottom channel at 30 μL/h (0.02 dyne/cm^2^) starting the day after cell seeding. 40-48 h after connection to flow, tumor cells (HCT116 H2B-GFP or DLD1 GFP *KRAS* mutant or DLD1 GFP *KRAS* WT) were seeded in the top channel at 2 × 10^4^ cells in 50 μL; 1.2 × 10^5^ cells cm^−^^2^. Cyclic, peristalsis-like membrane deformations (10% strain, 0.2 Hz) were initiated the day after tumor cell seeding using an electronic vacuum pump system (Emulate, Inc).

#### CRC-organoid-chip

Emulate Chip-S1 Stretchable Chips were activated by Emulate Reagents 1 and 2 (Emulate, Inc., ER-1 and ER-2) under UV light for 20 min at room temperature. The epithelial channel was coated with 250 μg/mL Matrigel (Corning, #356231) and the endothelial channel was coated with a mixture of 30 μg/mL Fibronectin (Corning #356008) and 200 μg/mL type IV collagen (Millipore Sigma #C5533) and incubated at 4 °C overnight. The chips were then warmed at 37 °C for 1 h and each channel was rinsed with PBS and corresponding media. HIMEC (RFP-labeled or unlabeled) were resuspended at 6.0 × 10^6^ cells/mL and seeded on the bottom channel. The chips were incubated at 37 °C, inverted for 2 h, and returned to normal orientation to allow HIMEC cell adhesion throughout the channel membrane. CRC tumor organoids were collected from BME by incubating in Gentle Cell Dissociation Reagent (STEMCELL Technologies, #07174) at 4 °C for 45–60 min, followed by dissociation with 50% TrypLE (Gibco; 12605-028) supplemented with 10 μM LY-27632 (Millipore Sigma, #5.09228.0001) and passed through a 40 μm cell strainer (Falcon, #352340). Organoids were resuspended in colon media and seeded on the top channel at 11-14.3 × 10^6^ cells/mL density. All chips were incubated overnight in static conditions at 37 °C, 5% CO_2_. The chips were then washed with fresh media and connected to Emulate portable pod modules filled with gas equilibrated medium, and continuous flow (30 μL/h) was initiated through top and bottom channels for 48 h. Cyclic conditions (10% strain, 0.2 Hz) were initiated after the formation of a monolayer, regarded as Day 0, and continued until the end of the experiment at Day 6.

### Barrier Function

To assess the barrier formation of each CRC organoid, 50 μg/mL Dextran (Cascade Blue, 3 kDa) (Invitrogen, #D7132) was added to the top channel, beginning on connection to flow (Day −2). The Dextran was replenished at every media change and the effluent from both channels was collected throughout the experiment (Days −2, −1, 0, 1, 2, 4, 6). The fluorescence from the effluent of both channels was measured using a plate reader (Biotek) and apparent permeability (P_app_) was calculated using the following formula:

$${P}_{{app}}=\,-\,\frac{{Q}_{R}* \,{Q}_{D}}{{S}_{A}* \left({Q}_{R}+\,{Q}_{D}\right)}* ln\left[1-\,\frac{{C}_{R,0}* ({Q}_{R}+\,{Q}_{D})}{({Q}_{R}* \,{C}_{R,0}+\,{Q}_{D}* \,{C}_{D,0})}\right]$$

where S_A_ is the surface area of the culture overlapping channel (0.17 cm^2^), Q_D_ & Q_R_ are the flow rates in the dosing channels (top epithelial) and receiving channel (bottom endothelial) respectively, in units of cm^3^/s, and C_R,0_ & C_D,0_ are the recovered concentrations in the receiving and dosing channels, respectively. For each donor, two independent experiments were conducted with duplicate or triplicate chips per condition.

### Immunofluorescence

#### Organoids

CRC organoids were seeded in 96-well plate with BME and cultured with colon media for 72 h. Each well was rinsed with PBS and fixed with 4% Paraformaldehyde (PFA) (ThermoFisher Scientific, #J61899.AP) at 4 °C on a rocker for 30 min. Each well was rinsed with 0.75% Glycine three times with 10 min incubation at room temperature followed by blocking buffer (5% bovine serum albumin (BSA), 0.2% Triton X-100, 0.04% Tween 20) for 2 h at room temperature. Organoids were stained overnight with primary antibody mouse anti-ZO-1 (1:250; Invitrogen #339100) in blocking buffer at 4°C. The next day, each well was rinsed with PBS three times with 20 min incubation at room temperature and stained with secondary antibody goat anti-mouse Alexa Fluor 647 (1:250; Invitrogen #A-21235) at room temperature for 90 min. After rinsing with PBS three times, organoids were stained with DAPI (1 μg/mL; Millipore Sigma, # D9542) for 10 min. Images were taken using an Olympus FV3000 confocal fluorescence microscope.

#### On-Chip

CRC Organoid-Chips were washed by flowing PBS through the endothelial and epithelial channels. The chips were fixed with 4% paraformaldehyde (ThermoFisher Scientific, #J61899.AP), incubated for 15 min, and permeabilized with 1% saponin. Blocking buffer of 2% bovine serum albumin (BSA) and primary antibodies were incubated overnight at 4 ^o^C before a 2 h incubation with secondary antibodies (1:500, Invitrogen, #A21428 and #A21235) diluted in blocking buffer. The primary antibodies used for the chip studies were mouse anti-ZO-1 Alexa Fluor 594 (1:100; Invitrogen #339194), rabbit anti-GABA (1:400; Millipore Sigma #A2052), rabbit anti-VE-cadherin (1:25; Abcam, #ab33168), and DAPI (Millipore Sigma, #D9542) was used to label all nuclei. Chips were imaged using the Perkin Elmer Operetta CLS High Content System.

#### Tissue slides

Formalin-fixed paraffin-embedded (FFPE) slides were incubated in a slide warmer for 1 h at 60 °C and further de-paraffinized by immersion in HistoChoice (Millipore Sigma, #H2779) for 3 rounds of 5 min each. To rehydrate, the slides were sequentially immersed, for 3 min each, 100% Ethanol, 100% Ethanol, 100% Ethanol, 95% Ethanol, 95% Ethanol, 70% Ethanol, 50% Ethanol, DI water, DI water. The slides were placed in a container with Antigen Retrieval Buffer (1 mM EDTA Buffer (Newcomer Supply #1056 A), 0.05% Tween 20), heated to boiling, and cooled for 30 min in buffer. Once cooled, the slides were incubated with 1 M Glycine for 30 min in a humified chamber and rinsed three times with 1x PBS. The slides were incubated with blocking buffer (2.5% Goat Serum (Vector Laboratories #S-1012-50), 0.05% Tween-20) for 1 h. Each slide was stained overnight with rabbit anti-GABA (1:400; Millipore Sigma A2052) and mouse anti-CK20 (1:100; Abcam, #Ab854 [KS20.8]) in blocking buffer at 4 °C followed by three washes with 1x PBS. The slides were incubated with goat anti-rabbit Alexa Fluor 647 (1:100; Invitrogen #A-21235) and goat anti-mouse Alexa Fluor 555 (1:100; Invitrogen, #A21235) in blocking buffer for 2 h, then rinsed with 1 X PBS three times. The slides were lastly stained with mouse anti-EpCAM FITC antibody (1:100; GeneTex, #GTX30708) in blocking buffer for 2 h followed by rinsing with 1 X PBS three times. Each slide was mounted with VECTASHIELD Antifade Mounting Medium (Vector Labs, #H1000-10) and sealed with a coverslip and clear nail polish. Images were on the Olympus VS120 Slide Scanner and quantified via ImageJ.

### RNAseq (Organoid, OOC, and tumor tissue)

#### RNA extraction from OOC

Endothelial HIMECs were removed and discarded via Trypsin from the bottom channel prior to collecting organoids. Organoids in the top channel were lysed using Lysis Buffer (Invitrogen, #46-6001) with 1% 2-mercaptoethanol (Millipore Sigma, #M3148) and vigorous pipetting. RNA was isolated using PureLink RNA Mini Kit (Invitrogen, #12183018 A) following manufacturer’s instructions.

#### RNA extraction from organoids and tumor tissue samples

RNA was isolated from organoids and tumor tissue using AllPrep DNA/RNA Mini Kit (Qiagen, #80204).

#### RNA-seq

Sequencing libraries were generated by SMARTer Stranded Total RNAseq Kit – Pico Input Mammalian V3 (Takara Bio, #634485) according to manufacturer’s instructions. Organoid and tumor tissue cDNA synthesis, sample quality assessment, cDNA library preparation, and sample sequencing were performed by the University of Southern California (USC) Genomics Core Facility (organoids and tumor tissue). Samples were sequenced on NextSeq 500 (Illumina). Read length was 75 bp. Organoids-on-chip cDNA synthesis, sample quality assessment, cDNA library preparation, and sample sequencing were handled by GENEWIZ from Azenta Life Sciences. Samples were sequenced on HiSeq 2500 (Illumina) rapid run flow cells. Read length was 150 bp.

#### Alignment and quality control

Quality of raw reads was evaluated using fastp. STAR was used to align reads against GENCODE v39 annotations (GRCh38). MultiQC was used to summarize statistics across all samples. Both duplication and alignment rate were used to select good-quality samples. Samples were further inspected for outliers using principal component analysis (PCA) and correlation. Blinded variance stabilizing transformed (VST) values were used as inputs for both PCA and correlation. Quality control metadata were visualized on the principal components and a heatmap of correlations by sample.

#### Differential expression and gene set enrichment analysis

DESeq2 was used to perform differential expression using a negative binomial model with a Wald test to determine significance. Default DESeq2 metrics were used to filter genes based on expression levels and calculate normalization factors. All visualizations of gene expression use values derived from a blind variance stabilizing transformation of raw counts. Volcano plots of results were generated using EnhancedVolcano with an adjusted *P* value threshold of 0.05 to indicate statistical significance and a foldchange threshold of +/− 2. Results were subsequently processed by gene set enrichment analysis using fgseaMultilevel from the fgsea package with all Hallmark gene sets. Summary visualizations of gene set enrichment results were generated by unsupervised selection of the 10 most significant gene sets in the positive and negative direction. Over-enrichment analysis was performed using DAVID functional analysis clustering (DAVID Functional Annotation Bioinformatics Microarray; https://david.ncifcrf.gov) using GO and KEGG gene sets. For this analysis, medium stringency was used (similarity term overlap = 3, similarity threshold = 0.5, group membership = 3, multiple linkage threshold = 0.5, and EASE = 1).

### Mass Spectrometry-based Metabolomics of CRC Organoid-Chip

#### Metabolite extraction

A 100 μL aliquot of each sample was extracted with 500 μL extraction solvent (80/20 Methanol/Water, −20 °C) supplemented with 10 μL “quench” internal standard mix (Cambridge Isotopes Inc. Metabolomics QC Kit, #MSK-QC2). The solution was briefly vortexed and sonicated for 1 min and then incubated for 1 h incubation at −20 °C. Samples were then centrifuged at 13,000 x *g* for 30 min at 4 °C. Without disturbing the pellet, 450 μL of supernatant were transferred to a new microcentrifuge tube and dried under vacuum centrifugation at room temperature (approximately 2 h). The dried samples were resuspended in 100 μL of resuspension solvent (50/50/0.1 (v/v) Water/Acetonitrile/Formic Acid) supplemented with 10 μL “resuspension” internal standard mix (Cambridge Isotopes Inc. Metabolomics QC Kit, #MSK-QC1).

#### Liquid Chromatography with Tandem Mass Spectrometry (LC-MS/MS)

Extracted metabolites were analyzed using an Agilent ultra-high performance liquid chromatography (UHPLC) system (1290 Infinity II HPLC) interfaced with an Agilent quadrupole time of flight mass spectrometer (Q-TOF 6545) equipped with an orthogonal Jet Stream Technology Electrospray Ionization (DUAL AJS-ESI) interface. Samples were separated by 2 different chromatographic separation methods using two analytical columns and conditions. The hydrophilic interaction liquid chromatography (HILIC) with Agilent Poroshell HILIC-Z column (2.1 × 100 mm, 2.7 μm) (Agilent InfinityLab Poroshell 120 HILIC-Z, #675775-924) used a two solvent gradient (Mobile Phase A: 10 mM Ammonium Acetate in 90/10 Water/Acetonitrile, pH 9.2, 5 μm Agilent InfinityLab Deactivator Additive; Mobile Phase B: 10 mM Ammonium Acetate in 90/10 Acetonitrile/Water, pH 9.2, 5 μm Agilent InfinityLab Deactivator Additive) at a flow rate of 0.5 mL/min. The column was equilibrated at 98% B for 3 min between runs. Subsequent to sample injection, the ratio was held for 3 min at 98% B, after which the solvent ratio was then reduced from 98% B to 50% B over 7 minutes and then brought back up to 98% B over 5 min. The second chromatography was reverse-phase liquid chromatography (RP-HPLC C18) with an Agilent Zorbax RRHD Eclipse Plus C18 column (2.1 × 50 mm, 1.8μm) (Agilent Zorbax RRHD Eclipse Plus C18, #959757-902), which used a two-solvent gradient (mobile phase A: water with 0.1% formic acid, B: methanol with 0.1% formic acid as mobile phases A and B, at a flow rate of 0.3 mL/min). The C18 column was equilibrated at 2% B, and a sample was injected and held for 3 min at 2% B, after which the solvent ratio was increased from 2% B to 98% B over 12 min, held at 98% B for 2 min, and then reduced back to 2% B over 2 min. For both HILIC and RP-HPLC C18 chromatographic separation, data were collected in positive and negative ion mode. The injection volume was 2 μL and the column temperature was set at 30 °C. Data were acquired from 50 to 1250 m/z at 1 spectra/s. The jet stream technology electrospray ionization (AJS-ESI) parameters were: gas temperature 290^o^ C (325° C for C18), drying gas 9 L/min, nebulizer 35 psi, fragmentor 125 V, sheath gas temperature 350° C, sheath gas flow 11 L/min at 1000 V nozzle voltage.

#### Metabolite data analysis

The LC-MS/MS data were processed by Agilent Mass Hunter Workstation Data Acquisition (.d) and analyzed by Agilent Mass Hunter Profinder for batch recursive feature extraction. For both targeted and untargeted analysis, spectral peak extraction was performed with a minimum peak height of 500 counts and a charge state of one. Retention time and mass alignment corrections were performed on the runs to remove non-reproducible signals. Feature identification was performed by matching their m/z values and retention times (when available) with in-house libraries of measured values for the IROA technologies MSMLS compound library. Metabolites that did not match our internal library were matched against the METLIN library or were listed with a predicted molecular formula.

Targeted feature extraction (including for GABA and the ^13^C_4_) was performed against a custom library of neurotransmitters using Profinder as described above. Features were exported to CSV for manual analysis by Excel.

### GABA Metabolite Measurements

For sample preparation, 1 μg/mL of GABA (Millipore Sigma #A2129) or ^13^C_4_ GABA (Cambridge Isotope Laboratories, Inc. #CLM-8666-PK) was added to the top channel inlet media and flown through the channel over the course of the six-day experiment. Media was replaced on day 3, and cells were harvested from the top channel of the CRC tumor chips and processed via mass spectrometry-based metabolomics described in the previous section. The intensity of the internal standard ^13^C_11_ Tryptophan was plotted as a control.

### Invasion Assay on-chip

Invasion was monitored and quantified as previously described [31, 32]. For GABA and GAD1 inhibition studies, the following drugs were perfused through the top channel starting on day 0. GABA (1 μg/mL or 10 μg/mL; Millipore Sigma #A2129) and 3-MPA (5 μM; Millipore Sigma #M5801). 3-MPA and GABA concentrations were sourced from previous studies examining GABA in tumor model systems [7, 33]. Inlet media with the GABA or GAD1 inhibitor was refreshed on day 3 of the experiment.

### RT-qPCR

To interrogate gene expression, RT-qPCR analysis was performed. Cellular RNA was extracted as outlined above (RNAseq), and cDNA was reverse transcribed using iScript Reverse Transcription Supermix (Bio-Rad, #1708841) following the manufacturer’s instructions. The cDNA was then amplified using iScript SYBR Green Master Supermix or iScript SsoAdvanced^TM^ SYBER Green Master Supermix (Bio-Rad; #1708880 or #1725271). Human *GAD1* PrimePCR primers were purchased from BioRad and the sequence for primers are as follows: GAPDH: F 5’-TCTGGTAAAGTGGATATTGTTG-3’, R 5’-GATGGTGATGGGATTTCC-3’; TFRC: F 5’-GTTGAATTGAACCTGGAC-3’, R: 5’-AAGTAGCACGGAAGAAGT-3’; E-Cadherin: F 5’-TTTGTACAGATGGGGTCTTGC-3’, R 5’-CAAGCCCACTTTTCATAGTTCC-3’; Snail: F 5’-ACCACTATGCCGCGCTCTT-3’, R 5’-GGTCGTAGGGCTGCTGGAA-3’; Ki67: F 5’- CTTTGGGTGCGACTTGACG-3’, R 5’- GTCGACCCCGCTCCTTTT-3’. Results were normalized to either GAPDH or TFRC expression for all experiments.

### Western Blot

Total protein was extracted and lysed in RIPA buffer with 1x Halt^TM^ protease and phosphatase inhibitor cocktail (Thermo Scientific Cat# 1861282) and protein concentrations were determined using the Pierce^TM^ BCA Protein Assay Kit (Thermo-Fisher, #23225). 10 μg of protein lysates were boiled in SDS loading buffer (Life Technologies, #B007). Protein samples were loaded and fractionated on a 4–12% Bis-Tris gel (Life Technologies, #NW04122BOX) using 1xBolt^TM^ MES SDS Running Buffer (Thermo-Fisher, #B0002) and then transferred to a polyvinylidene difluoride membrane. The membrane was blocked in 5% bovine serum albumin (BSA) in Tris-buffered saline with 0.1% Tween-20 (TBS-T buffer), and primary antibodies against total protein were used. Secondary antibody (mouse IgG-HRP; 1:5000; Cytiva, #NA931; rabbit IgG-HRP; 1:10,000; Cytiva #NA934) was visualized using an enhanced chemiluminescent horseradish peroxidase substrate. A protein ladder was used to determine molecular mass, and ImageJ was used to measure the intensity of the blot. The primary antibodies used for the HCT116 H2B-GFP GAD1 knockdown validation were mouse anti-GAD1 (1:500; Santa Cruz Technology, Inc., #sc-28376) and rabbit anti-GAPDH (1:1000; Cell Signaling Technology, #5174).

### Human Tumor GAD1 Data Analysis

RNA-seq data from The Cancer Genome Atlas (TCGA) colonic adenocarcinoma database (TCGA-COAD) was extracted from the cBioportal for Cancer Genomics (www.cbioportal.org) and RNA-seq data (GSE39582) [34] as extracted from the Gene Expression Omnibus (GEO) (https://www.ncbi.nlm.nih.gov/geo/). CRC cases were selected based on any *KRAS*, *NRAS*, or *BRAF* mutations and any treatment status was included. It is important to note that, to enhance the sample size of patient tumors for this study, we incorporated tumors irrespective of their treatment history. The expression of *GAD1* in the *KRAS* mutant tumors versus *KRAS* WT tumors in the primary colon tumor was analyzed. All *KRAS*, *NRAS*, or *BRAF* mutant CRC tumors were stratified based on *GAD1* expression, with “high” and “low” *GAD1* expression designated as above or below the median expression for the cohort (TCGA cohort: median mRNA expression z-scores relative to all samples = 0.3; GSE39582 cohort: median normalized mRNA expression = 3.4). CRC cases were selected based on any KRAS, NRAS, or BRAF mutations and any treatment status was included. The prognosis of each group was examined using Kaplan-Meier survival estimators and significance was assessed using a log-rank (Mantel-Cox test). A Hazard Ratio (HR) with 95% confidence interval (CI) of the ratio was calculated using a log-rank test.

### DepMap Data Analysis

Gene expression (RNA-seq) and MetMap data was accessed and downloaded from the DepMap website (https://depmap.org/portal/) on December 2023-April 2024. Linear regression analysis was performed in and Pearson correlation coefficient was calculated by GraphPad Prism 9 software.

### Sample Size, Quantification, and Statistical Analysis

#### Chip experiments

Sample size calculations were based on preliminary data from triplicate assays across two experimental days. These experiments yielded intra-day standard deviation estimates of 0.12 and 0.10 for the (natural) logarithmic invasion ratio and logarithmic cell count, respectively (approximately 12% and 10% in absolute terms). The logarithmic transform successfully normalizes variance both for invasion ratios and cell counts. We designed all experiments to detect a 50% change (in invasion ratio or cell count) with at least 80% power at *p* = 0.05 significance. Experimental factors will be randomly assigned to chip location within the culture module to preclude systematic bias. To detect a difference in invasion ratio and growth rate between experimental conditions, four replicates will supply 98% and 92% power, respectively, using a two-sided t-test to detect a 50% change.

Unless otherwise noted, all experiments were performed with 2–3 chips per condition and repeated independently at least 2 times. For all Figures, the datapoints on the graphs represent the replicates used for statistics. For on-chip invasion data (Figs. 2, 5, or S6) or GABA measurements (Fig. 4), each chip represented a sample for statistical analysis (*N* = 4–6). For qPCR analysis from cells harvested from chip (Fig. 5) and GABA measurements from the tumor tissue (Fig. 3), stats were performed on the mean of the technical replicates of each group (*N* = 2 for qPCR and *N* = 3 for GABA measurements). Note: for qPCR analysis, *n* = 5 chips were pooled from two biological replicates due to limited material available for signal detection. Analysis of variance (ANOVA) and t-tests with Šídák’s multiple comparison test as indicated in the figure legends were performed using GraphPad Prism 9 software, with the *P*-value < 0.0001: **** *P*-value < 0.001: *** *P*-value < 0.01: ** *P*-value < 0.05: *, as noted in all figure legends. Unless otherwise noted, individual data points are reported and shown in all figures, and the mean with standard error of the mean (SEM) is represented.

## Results

### GABAergic changes in colorectal cancer are linked to patient outcomes

**Fig. 1:**
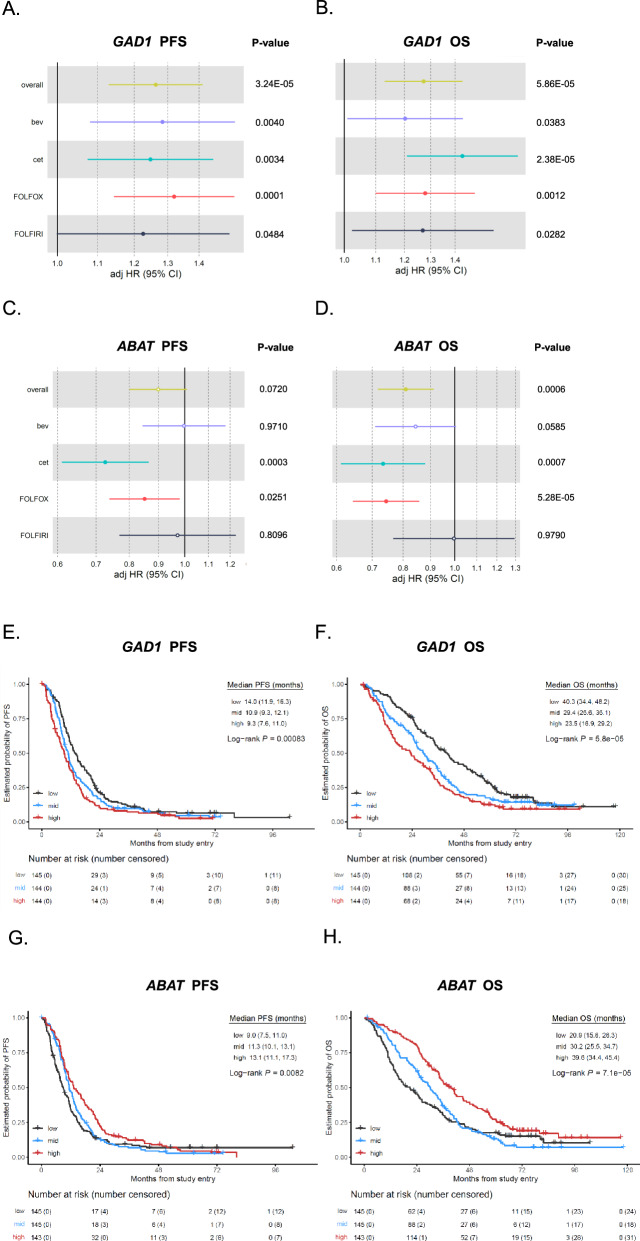
Association between GABA pathway gene expression and patient outcomes in the CALGB/SWOG 80405 trial. Forest plots show the association of *GAD1* tumor expression as a continuous variable with PFS (**A**) and OS (**B**) across treatment arms; Forest plots show the association of *ABAT* tumor expression with PFS (**C**) and OS (**D**) across treatment arms. Kaplan-Meier curves show PFS (**E**) and OS (**F**) stratified by *GAD1* tumor expression tertiles (T1 low, T2 middle, and T3 high) in the overall population of the CALGB/SWOG 80405 trial. The statistical comparison is performed for T3 vs T1. Kaplan-Meier curves show PFS (**G**) and OS (**H**) stratified by *ABAT* tumor expression tertiles in the overall population of the CALGB/SWOG 80405 trial. The statistical comparison is performed for T3 vs T1. *P* values are based on log-rank test for PFS and OS in the univariate categorical analysis, and likelihood ratio test in the univariate continuous and multivariable Cox proportional hazards regression model. bev bevacizumab, cet cetuximab, CI confidence interval, HR hazard ratio, OS overall survival, PFS progression-free survival.

To evaluate the clinical significance of GABA pathway-related genes in CRC, we analyzed the associations between GABA-related gene expression in the tumor and survival outcomes in patients with metastatic colorectal cancer (mCRC) enrolled in a large, randomized first-line phase III trial, CALGB/SWOG 80405 [35]. Patient demographics and tumor characteristics of the CALGB/SWOG 80405 cohort are summarized in Table S1. Overall, among the genes individually associated with survival outcomes, high *GAD1* was associated with shorter progression-free-survival (PFS) (hazard ratio [HR] per 1 standard deviation increase in *GAD1* expression: 1.26, 95% confidence interval [CI] 1.13, 1.41, multivariable *P* = 3.2E−05) and overall survival [OS] (HR 1.27 [1.13, 1.43], multivariable *P* = 5.9E-05) across treatment arms when gene expression was evaluated as a continuous variable (Fig. 1A, B). This effect was consistent in targeted treatment (bevacizumab or cetuximab) and chemotherapy backbone (FOLFOX or FOLFIRI) subgroups. Conversely, high *ABAT* (encoding for the aminotransferase responsible for GABA catabolism) was associated with longer OS (HR 0.81 [0.72, 0.91], multivariable *P* = 6E−04) (Fig. 1C, D). This association was stronger in patients treated with the anti-EGFR cetuximab or with FOLFOX chemotherapy, where high ABAT was also associated with longer PFS (*P* = 0.0003 and *P* = 0.025), whereas no significant associations were found in patients treated with the anti-VEGF bevacizumab or with FOLFIRI. Categorization of gene expression into tertiles (T1 low, T2 middle, and T3 high) highlighted a PFS benefit of 14.0 vs 9.3 months (log-rank *P* = 0.00083, multivariable *P* = 0.0025) and 13.1 vs 9.0 months (log-rank *P* = 0.0082, multivariable *P* = 0.51) for *GAD1* T1 vs T3 and *ABAT* T3 vs T1, respectively, in the overall trial population (Fig. 1E, G). The OS benefit was 40.3 vs 23.5 months (log-rank *P* = 5.8E−05, multivariable *P* = 0.0023) and 39.6 vs 20.9 months (log-rank *P* = 7.1E−05, multivariable *P* = 0.015) for *GAD1* T1 vs T3 and *ABAT* T3 vs T1, respectively (Fig. 1F, H). Complete results for all tested genes are presented in Table S2. Overall, these results suggest that increased GABA production (high *GAD1*) and reduced GABA catabolism (low *ABAT*) are associated with worse outcomes for mCRC patients.

### Relationship between GAD1 and metastatic potential

**Fig. 2:**
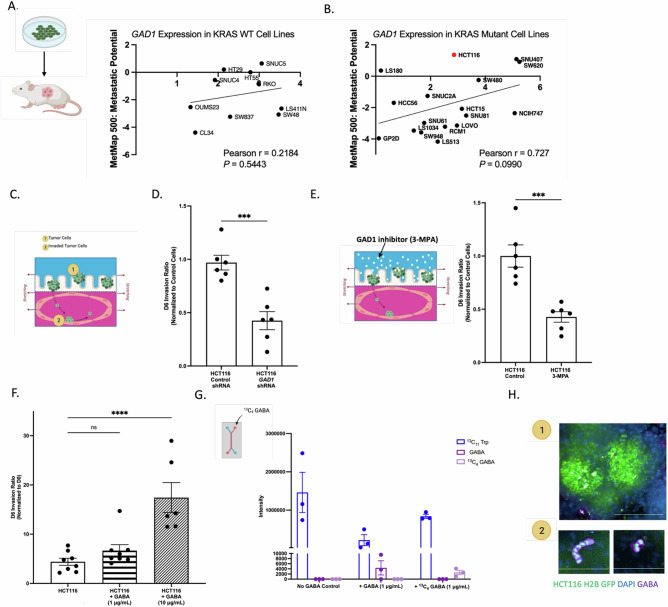
GAD1 is associated with CRC metastatic potential. **A** *GAD1* expression of *KRAS* wild-type (WT) CRC cell lines plotted against their metastatic potential in mice (DepMap). **B** *GAD1* expression of *KRAS* mutant CRC cell lines plotted against their metastatic potential in mice (DepMap). **C** Schematic of OOC model of tumor cell lines invading from the top channel into the bottom endothelial channel. **D** On-chip tumor invasion of *GAD1* knockdown HCT116 tumor cells, normalized to control shRNA. Data was analyzed using an unpaired t-test. N = 6 chips; ***P = 0.0005. **E** On-chip tumor invasion of HCT116 tumor cells treated with GAD1 inhibitor 3-MPA (5 μM), normalized to vehicle control. Data was analyzed using an unpaired t-test. N = 6 chips; *** *P* = 0.0006. **F** On-chip tumor invasion of HCT116 tumor cells with increasing concentrations of exogenous GABA. Data was analyzed using a one-way ANOVA with Šídák’s multiple comparison test. *N* = 6–8 chips; ****P < 0.0001. **G** Intracellular [^13^C_4_]GABA or unlabeled GABA was measured via mass spectrometry-based metabolomics in the HCT116 tumor-chips after the addition of exogenous GABA for six days. *N* = 3 chips. The intensities of the internal standard ^13^C_11_ Tryptophan (Trp) were plotted as a control. **H** On-chip GABA staining in the top channel (1) representing non-invaded HCT116 tumor cells, and in the bottom channel (2) capturing invaded HCT116 tumor cells. Scale bars represent 200 μm in the top channel image and 100 μm in the bottom channel images. Individual data points are shown and mean ± SEM are represented. All schematics were made in or are from BioRender.

Considering the clinical importance of high *GAD1* expression and its association with worse survival outcomes in CRC, we analyzed publicly available datasets to further investigate the link between *GAD1* and tumor progression. We utilized the MetMap 500 dataset published by DepMap (https://depmap.org/portal), which quantifies the ability of cell lines to form metastases in mice. We found an association between higher *GAD1* expression and increased metastatic potential, which was further enhanced when stratifying cell lines by *KRAS* mutational status (Fig. 2A, B).

To further investigate this relationship, we employed a human-relevant OOC model system that enables functional studies and provides mechanistic insights into tumor cell intravasation, an early step in the metastatic cascade [31, 32]. The model contains epithelial and endothelial compartments separated by a porous membrane and incorporates fluid flow and peristalsis-like motions to recreate the mechanical forces found within the gut. Initially, we chose to investigate the role of GAD1 on tumor cell invasion using the HCT116 tumor cell line (denoted by a red dot in the MetMap plots), a *KRAS* mutant cell line with high *GAD1* expression and high metastatic potential. We have previously characterized the invasive potential of the HCT116 cell line in our OOC model, quantifying tumor cells invading from the top, epithelial channel through an extracellular matrix (ECM) coated-membrane, and into the bottom, endothelial channel using live cell imaging [31, 32] (Fig. 2C). When *GAD1* expression was reduced using shRNA knockdown or GAD1 activity was pharmacologically inhibited via 3-mercaptopropionic acid (3-MPA) in HCT116 tumor cells, we observed reduced intracellular GABA concentrations and reduced tumor cell intravasation into the endothelial compartment (Figs. 2D, E, S1). Furthermore, introducing exogenous GABA to the epithelial compartment within the OOC model, resulted in increased tumor cell intravasation (Fig. 2F). We confirmed active uptake of GABA by the HCT116 cells by quantifying intracellular levels of ^13^C_4_-labeled GABA using mass spectrometry (Fig. 2G). We also confirmed strong GABA staining in the invaded HCT116 tumor cells compared to the non-invaded cells, suggesting a role for tumor-derived GABA in the intravasation process (Fig. 2H).

### Characterization of tumor-derived GABA in CRC tissues

**Fig. 3:**
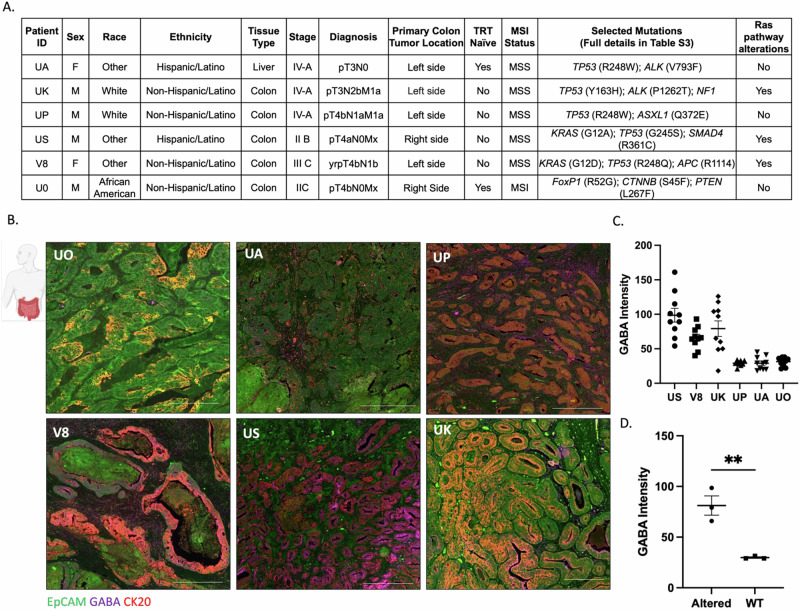
Tumor-derived GABA is increased in Ras altered tumors. **A** Tumor characteristics and patient demographics. **B** Representative 10x immunofluorescence images of the 6 tumors stained for EpCAM (green), CK20 (red), and GABA (purple). Scale bars represent 500 μm for all tumors except for UK at 200 μm, due to the different tissue sizes. **C** GABA intensity quantification of *N* = 10 regions that were CK20+ tumor areas/patient. **D** Average GABA intensity grouped by Ras signaling. *N* = 3 patients/grouping; **P = 0.0057. Data was analyzed using an unpaired t-test. All data are shown as mean ± SEM. All schematics were made in or are from BioRender.

**Fig. 4:**
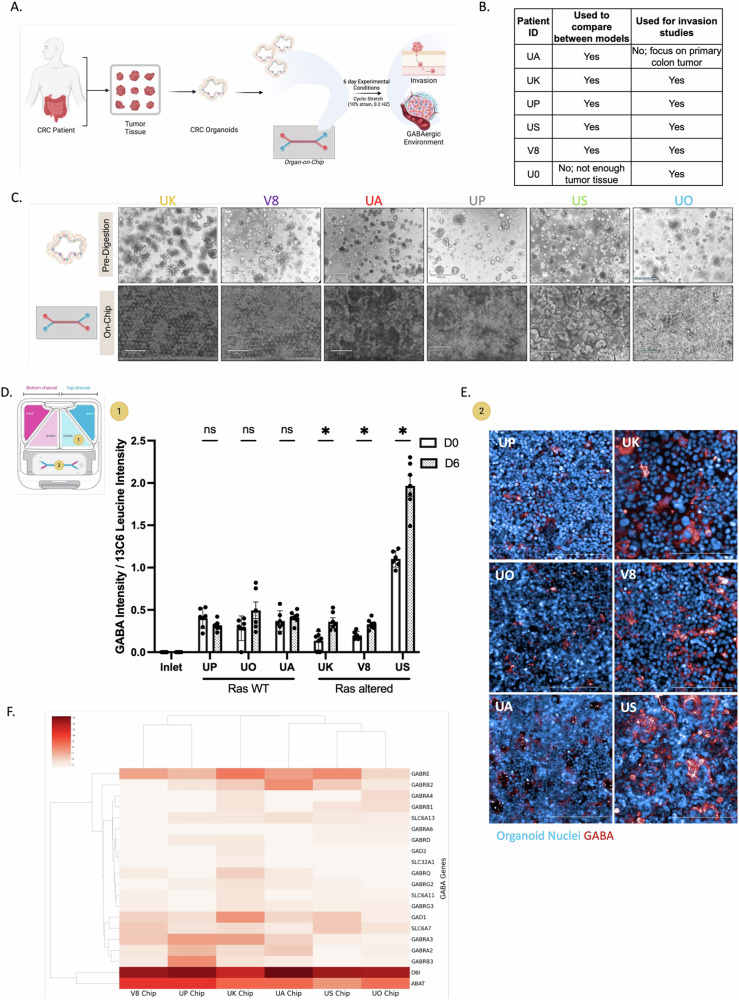
Development of patient-derived organoid-chips to model a GABAergic TME. **A** CRC organoids were derived from patient tumor tissues and were seeded onto OOC devices with fluid flow and peristaltic mechanical forces. The GABAergic on-chip environment (GABA-related gene expression and GABA levels) and the invasive properties of the organoid-chips were characterized. **B** Six patient-derived organoids were used for various analyses on-chip. **C** Brightfield images of patient-derived CRC organoids in culture prior to digestion (top panel) and after 6 days on-chip (bottom panel). Scale bars for the pre-digested organoids represent 500 μm and scale bars for the organoids on-chip represent 210 μm. **D** Effluent from the epithelial channel of the patient-derived organoids was collected over time (day 0 (D0) and day 6 (D6)) and metabolites were measured via mass spectrometry-based metabolomics (yellow 1 indicated in schematic). GABA intensity was analyzed from extracted metabolites *N* = 6 chips per timepoint per patient and reported as intensity normalized to a ^13^C_6_ Leucine internal standard; *P = 0.0312. Data was analyzed using multiple paired t-tests. All data are shown as mean ± SEM. **E** GABA present in the top channel of the organoid-chips was measured via immunofluorescence at the end of the experiment on day 6 (yellow 2 indicated in schematic). Representative immunofluorescence images of GABA staining (purple) on-chip. Scale bars represent 200 μm. **F** Expression of GABAergic-related genes compared across the organoid-chips from RNAseq analysis.

Previous studies, along with our clinical trial data, suggest the presence of GABA in the TME of some CRC tumors. Building on this data and the cell line results presented above, we examined GABA levels in CRC tumor tissues by selecting six tumors from our CRC tumor biorepository. These samples were chosen to ensure representation across key factors such as sex, race/ethnicity, cancer stage, and mutational status (as shown in Fig. 3A and Table S3), and the availability of viable tissues for patient-specific OOC development described in Fig. 4. We grouped any mutations within the Ras pathway as Ras “mutant,” to represent altered Ras signaling. While the UK tumor did not have any mutations in *KRAS*, we did identify a mutation in *NF1*, which is a negative regulator of Ras pathway activation. Tumor tissues from the six patients were stained for GABA (purple) and for EpCAM (green) and CK20 (red) to delineate tumor cells (Figs. 3B and S2). Quantification of the GABA signal in the tumor cells showed significant patient heterogeneity (Fig. 3C). Comparing GABA expression levels from tumors with altered Ras signaling (US, V8, and UK) to tumors with unaltered Ras signaling (UA, UP, and U0) resulted in significantly higher GABA in the tumor regions with altered Ras signaling (Fig. 3D).

### Development of patient-specific CRC organoid-chip models to study GABA and inter-tumor heterogeneity

To investigate the functional role of GABA in heterogeneous CRC tumors, we assessed its effect through patient-derived organoid-chip models. We were motivated to develop organoid-chip models derived from the six tumor tissues mentioned above, given the significant advantages of our OOC model in replicating key aspects of the CRC TME, including its microfluidic capabilities, the influence of peristaltic forces, and the ability to monitor invasion events (Fig. 4A, B). To create a patient-derived CRC organoid-chip, tumor organoids were introduced into the ECM-coated top channel, while human intestinal microvascular endothelial cells (HIMECs) were seeded into the ECM-coated bottom channel, separated by a porous membrane. Approximately one day after cell seeding (considered Day -2), we initiated flow at a specific rate of 30 μL h^−1^. To mimic physiological peristalsis, we introduced mechanical stretching at 10% deformation and 0.2 Hz approximately 48 h later. The values for peristalsis were selected based on clinical measurements of the intestine and previous work performed in normal intestine-chips [36, 37]. These experiments continued for an additional 6 days, as depicted in the experimental timeline in Fig. S3.

Each patient-specific organoid-chip displayed robust adherens junction proteins (zonula occludens-1; ZO-1 and VE-Cadherin) and formed intact intestinal barriers by Day 0 (calculated by Apparent Permeability, P_app_, using Cascade Blue Dextran), which was consistently maintained for over one week, as indicated in Fig. S4A–C. RNAseq analysis of our model system revealed significant variance among patients as expected, which is illustrated by the principal component analysis (PCA) in Fig. S5A. We then assessed the number of differentially expressed genes (DEG) (*P* < 0.05) in three key patient-matched comparisons: organoid alone vs tumor tissue, organoid-chip vs tumor tissue, and organoid-chip vs organoid alone, involving a cohort of 5 patients (Fig. S5B). The lower number of DEGs in the organoid-chip vs tumor comparison (*n* = 4168) than in the organoid vs tumor comparison (*n* = 5716) suggests that the organoid-chip model more closely reflects the transcriptomic profile of the tumor tissue than the organoid model (Fig. S5C). Genes that were up- or down-regulated in the organoid or organoid-chips were enriched in processes related to protein synthesis and metabolism. Conversely, genes with elevated expression levels in the tumor tissues displayed enrichment in ECM components and organization, cell adhesion, and various aspects of the immune system (Fig. S5D).

Moreover, when we compared key genes related to colorectal cancer progression between the organoid-chips and matched tumor tissues, there were limited differences in gene expression (Fig. S5E). The significantly different genes (p_adj_<0.05) identified were related to proliferation and apoptosis (e.g., *cyclin D; CCND1, BCL2, BAD*) and PI3K signaling pathways, which may be related to the media composition for cultivating organoids and the known differences in proliferation rates of in vitro model systems. Overall, the organoid-chip model mimics important aspects of CRC biology, including inter-tumor patient heterogeneity. We therefore leveraged this model to characterize the GABAergic environment and investigate its involvement in tumor progression (Fig. 4A–C).

First, we sampled the top channel effluent over the course of the experiment and performed a targeted mass spectrometry-based metabolomics analysis. While we observed variable amounts of GABA present across patient organoid-chips models, all had more GABA in the effluent than detected in the inlet media (Fig. 4D). Additionally, the US organoid-chips, which exhibited the highest GABA levels, showed a fivefold increase in GABA concentration in the top channel containing the tumor cells compared to the bottom channel, suggesting that the GABA was derived from the tumor cells (Fig. S6). Interestingly, we identified a significant increase in GABA levels over time (D0 to D6) for the three organoid-chips derived from altered Ras tumors (UK, V8, US), which was also confirmed by on-chip immunofluorescence staining for tumor-derived GABA (D6) (Fig. 4E). In addition, we confirmed expression of GABA-related genes, particularly *GAD1*, as measured by RNAseq of the tumor organoids cultured on-chip for six days (Fig. 4F). Among an extended panel of neurotransmitter-related genes, GABA-related genes were some of the most highly expressed (Figure S5F).

### Inhibiting GAD1 reduces invasion in tumor cells with altered Ras signaling on-chip

**Fig. 5:**
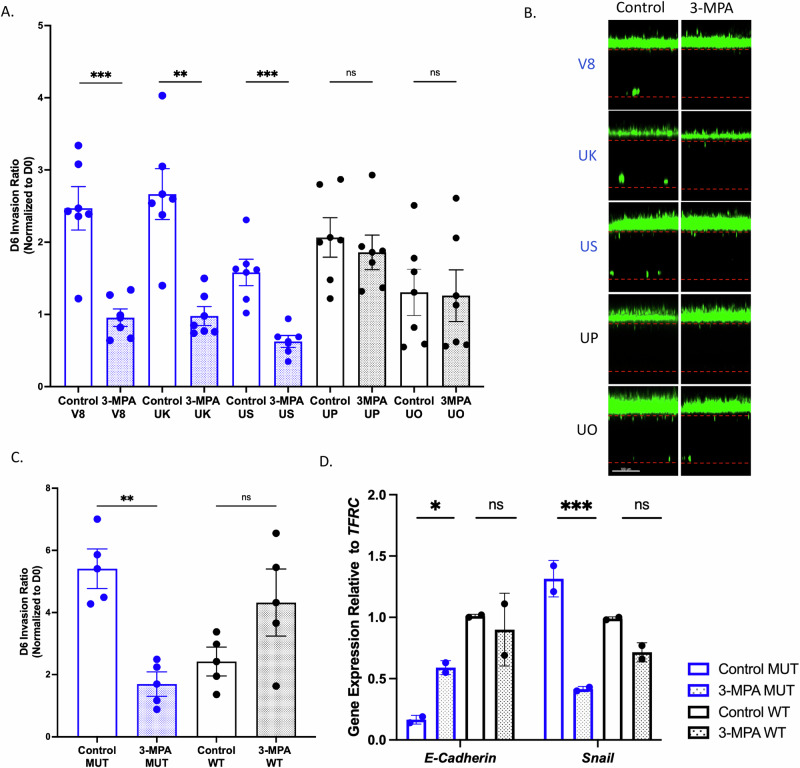
GAD1 inhibition reduces tumor cell invasion in tumors with altered Ras signaling. **A** On-chip invasion of organoids with or without 3-MPA treatment. N = 6 chips; UK**P = 0.0011; V8***P = 0.0009; US***P = 0.0008. Blue coloring indicates Ras altered organoids and black coloring indicates Ras WT organoids. Data was analyzed using an unpaired t-test. All data are shown as mean ± SEM. **B** Representative images of invasion on-chip with and without 3-MPA treatment. Scale bar represents 100 μm. **C** On-chip invasion of DLD1 isogenic *KRAS* cell lines (*KRAS* mutant or *KRAS* WT) with or without 3-MPA treatment. N = 5 chips; **P = 0.001. Data was analyzed using a one-way ANOVA with Šídák’s multiple comparison test. All data are shown as mean ± SEM. **D** *E-cadherin* and *Snail* gene expression of DLD1 isogenic *KRAS* cell lines with or without 3-MPA treatment. Tumor cells from the top channel of 5 chips across two biological replicates were harvested for qPCR. Circles on the graph represent the mean of 3 technical replicates. *N* = 2 biological replicates for statistical analysis; E-cadherin*P = 0.0493; *Snail* ***P = 0.0009. Data was analyzed using a one-way ANOVA with Šídák’s multiple comparison test. All data are shown as mean ± SEM. All schematics were made in or are from BioRender.

To test the hypothesis that tumors with altered Ras signaling are utilizing GABA signaling for cancer progression, we pharmacologically inhibited GAD1 with 3-MPA, measured on-chip tumor cell intravasation, and harvested cells for gene expression analysis. In a cohort of five patient-derived organoid-chips, those with altered Ras signaling (V8, UK, US) showed reduced tumor cell invasion in the presence of GAD1 inhibition, while those with WT Ras signaling (UP and UO) did not respond to GAD1 inhibition (Fig. 5A, B). As shown in Fig. S7, there were no significant differences in Day 6 tumor cell numbers between treatment and control chips in the top channel, suggesting GAD1 inhibition is not targeting proliferation in this model, but rather the invasive potential. This was further confirmed using isogenic DLD1 *KRAS* mutant and wildtype tumor cell lines, where we found that 3-MPA reduced on-chip invasion only in the *KRAS* MUT cell line (Fig. 5C). Tumor cell numbers on Day 6 were unchanged between treated and untreated conditions, and 3-MPA did not change Ki67 gene expression in either the *KRAS* mutant or wildtype cell lines (Fig. S7). Furthermore, we observed increased *E-Cadherin* expression and decreased *Snail* expression (Fig. 5D) when the DLD1 *KRAS* mutant cells were treated with 3-MPA, suggesting changes to adhesion molecules associated with decreased invasive potential. This was not observed in the DLD1 *KRAS* wildtype cell line.

### Elevated GAD1 expression is associated with poor prognosis in CRC patients with Ras altered tumors

**Fig. 6:**
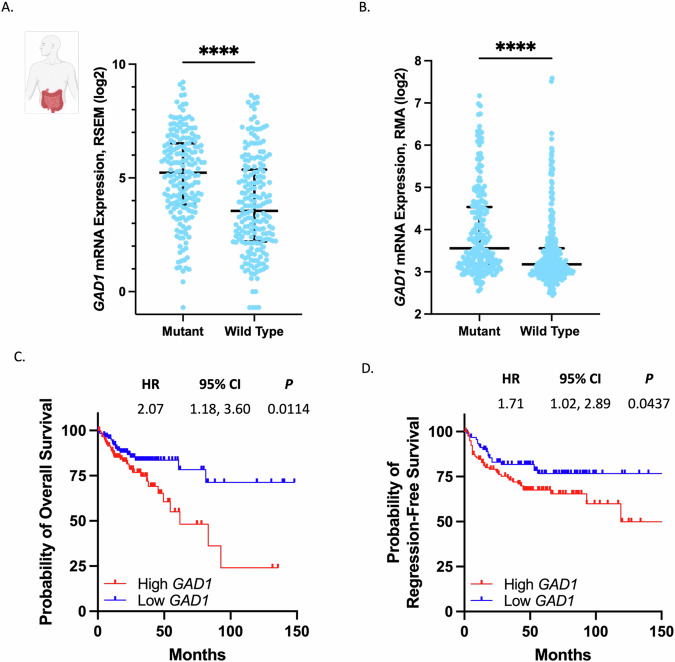
Higher *GAD1* expression is associated with worse outcome in Ras mutant patients. **A** *GAD1* mRNA expression from TCGA in primary colon cancer tumors. N = 196 patients with *KRAS*, *NRAS*, or *BRAF* mutant tumors; N = 201 patients with *KRAS*, *NRAS*, or *BRAF* wildtype tumors. Individual data points are shown and the median with interquartile range is represented. Data was analyzed with an unpaired t-test; ****P < 0.0001. **B** *GAD1* mRNA expression from the Gene Expression Omnibus (GEO), accession GSE39582, in primary colon cancer tumors. N = 220 patients with *KRAS* or *BRAF* mutant tumors; N = 276 patients with *KRAS* or *BRAF* wildtype tumors. Individual data points are shown and the median with interquartile range is represented. Data was analyzed with an unpaired t-test; ****P < 0.0001. **C** Kaplan-Meier curve with univariate analysis of the survival of patients with *KRAS*, *NRAS*, or *BRAF* mutated CRC tumors based on high versus low expression of *GAD1* (defined as above or below the median *GAD1* mRNA expression z-score of 0.3). Data was extracted from the TCGA. N = 254 patients. Data was analyzed using a log-rank (Mantel-Cox test) and a hazard ratio (HR) with 95% confidence interval (CI) is reported. **D** Kaplan-Meier curve with univariate analysis of the survival of patients with *KRAS* or *BRAF* mutated CRC tumors based on high versus low expression of *GAD1* (defined as above or below the median *GAD1* mRNA expression of 3.4). Data was extracted from the GEO, accession GSE39582. N = 217 patients. Data was analyzed using a log-rank (Mantel-Cox test) and a hazard ratio (HR) with 95% confidence interval (CI) is reported. All schematics were made in or are from BioRender.

A subsequent analysis of *GAD1* expression in two CRC patient samples sourced from The Cancer Genome Atlas (TCGA) (through the cBioPortal database) and a previously published study sourced from the Gene Expression Omnibus (GEO) [34] indicated a noteworthy increase in *GAD1* expression in primary colon tumors carrying *KRAS*, *NRAS*, or *BRAF* mutations, when compared to their *KRAS* wild-type counterparts (P < 0.0001), shown in Fig. 6A, B. Moreover, elevated *GAD1* expression in *KRAS*, *NRAS*, or *BRAF* mutant colon tumors is indicative of a less favorable prognosis (*P* = 0.0114 in the TCGA cohort and *P* = 0.0437 in the GSE39582 cohort), as illustrated in Fig. 6C, D.

In summary, our study integrates patient data with a patient-derived organoid-chip model to demonstrate that CRC tumors exhibiting high *GAD1* expression, which leads to elevated tumor-derived GABA levels, are linked to poorer patient outcomes. Additionally, we show that targeting GAD1 can effectively reduce tumor cell invasion, particularly in tumor cells with dysregulated Ras signaling.

## Discussion

In pathological conditions such as CRC, there is often dysregulation of neurotransmitters, which can impact intestinal function and homeostasis [4]. Throughout CRC progression, both cancer cells and neurons within the enteric nervous system of the intestine release neurotransmitters that can influence signaling pathways, potentially promoting the survival and aggressiveness of cancer cells [38]. However, understanding the mechanistic role of neurotransmitters in cancer progression and connecting neurotransmitter signaling in the TME with patient outcomes has been limited. Our primary objective was to utilize a MPS to replicate key features of the patient CRC TME and gain a mechanistic understanding of clinical trial data linking a GABAergic TME with worse patient outcomes. The results of the CALGB/SWOG 80405 clinical trial presented here build upon previous studies that have identified upregulated *GAD1* and downregulated *ABAT* in CRC tissue as compared to normal colon tissue [7, 39, 40].

To begin to understand the role of tumor-derived GABA in cancer progression, specifically in tumors with altered Ras signaling, we utilized publicly-available datasets that combined cancer cell line RNAseq data and the metastatic potential in mouse xenografts (DepMap) [41]. This work implicated increased metastatic potential of *KRAS* mutant CRC cell lines with high *GAD1* expression. Utilizing our tumor-on-chip model system, we hindered the tumor cells’ ability to produce GABA via genetically knocking down or pharmacologically inhibiting GAD1 and showed a reduction in tumor cell invasion. Previous studies have shown that inhibiting GAD1 reduces metastatic capabilities in mice by reducing CRC tumor cell proliferation and immune suppression [7]. In our model, we did not observe significant differences in proliferation, However, we identified changes in invasive properties, particularly in *KRAS* mutant cell lines, underscoring the additional insights MPS’s can offer. Findings in other cancer types have also demonstrated a link between GABAergic characteristics and metastasis [42, 43]. In breast and melanoma brain metastases, elevated GAD1 expression contributes to metabolic adaption that facilitates metastatic outgrowth [44].

We further validated the connection between altered Ras signaling and a GABAergic TME by analyzing CRC patient tissues from our biobank, along with data from two publicly available CRC cohorts. We created organoid-chip models utilizing patient-derived tumor organoids capable of maintaining patient diversity and more closely replicating crucial properties of the CRC tumor, including key pathways and genes related to CRC. This OOC model system provides a valuable tool for investigating various stages of metastasis in a personalized manner, which is a challenging aspect to replicate in a laboratory setting [45–47]. It’s important to acknowledge that achieving a complete replication of in vivo tumors when working with 3D in vitro models is impractical. The focus, instead, is on capturing the critical features most relevant to the intended context of use. For our study, it was crucial to replicate the epithelial-endothelial tissue interface to capture early metastatic events and incorporate patient-derived organoids to enable the study of inter-patient heterogeneity. We also identified potential gaps in our model by conducting an over-enrichment analysis that shed light on elements that were noticeably absent in our organoid-chips when compared to the matched tumor tissues. As expected, we observed a lack of immune cell signatures and certain ECM components; however, our model is adaptable, allowing for future modifications to include immune and other stromal cells or incorporate patient-specific tumor ECM. Given that GABA is an important signaling molecule within the immune TME landscape [7, 9], this represents an important area for follow-up research. For example, GABA secreted by B cells and tumor cells has been shown to suppress T cell activity and modulate macrophage polarity [9].

In summary, our study has provided valuable insights into the influence of GABA signaling on tumor cell behavior using an organoid-chip model of CRC. By mimicking key features of the TME and incorporating patient-specific elements, we have revealed interesting patterns in GABAergic properties in altered Ras tumors. This work is, to our knowledge, the first to connect altered Ras signaling with increased *GAD1* expression and GABA levels in CRC. Although the link is associative, the known poor prognosis of patients with *KRAS* mutations [48–50] and the pressing need for improved therapies highlight the importance of future mechanistic investigations into GABAergic and Ras signaling. Previous neuroscience work has shown that hyperactive Ras/MAPK pathways in neurons resulted in altered GABAergic signaling, including elevated *GAD1* expression [51] and GABA secretion [52]. Additionally, our identification of the role of GAD1 in cancer cell invasion suggests potential implications for CRC progression and targets for drug development. These findings enhance our understanding of cancer biology and underscore the versatility of the OOC system as a tool for exploring complex and heterogeneous interactions in cancer research.

### Limitations of the Study

CRC is a complex and diverse disease. Although we have developed organoids-on-chips from samples of six different patients with varying tumor characteristics, a more comprehensive analysis is required before confidently asserting subtype-specific findings, particularly in establishing a causal relationship between Ras and GAD1. This will also include validating GAD1 pharmacological inhibition using CRISPR/Cas9 to rule out potential off-target effects. Moreover, further research is essential to comprehend how tumor cells respond to dynamically changing and potentially patient-specific physical TME. Prior studies have observed the migration of CRC cancer cells along enteric neurons [53]; therefore, including the incorporation of enteric neurons to the OOC system would be an interesting follow-up study and relevant to the GABAergic investigations.

## Data and materials availability

All data are available in the main text or the supplementary materials.

## Supplementary information

Supplemental Material Combined

## References

[1] Berger MF, Mardis ER. The emerging clinical relevance of genomics in cancer medicine. Nat Rev Clin Oncol. 2018;15:353–65. 29599476 10.1038/s41571-018-0002-6 PMC6658089

[2] Stratton MR, Campbell PJ, Futreal PA. The cancer genome. Nature. 2009;458:719–24. 19360079 10.1038/nature07943 PMC2821689

[3] Mittal R, Debs LH, Patel AP, Nguyen D, Patel K, O’Connor G, et al. Neurotransmitters: the critical modulators regulating gut-brain axis. J Cell Physiol. 2017;232:2359–72. 27512962 10.1002/jcp.25518 PMC5772764

[4] Battaglin F, Jayachandran P, Strelez C, Lenz A, Algaze S, Soni S, et al. Neurotransmitter signaling: a new frontier in colorectal cancer biology and treatment. Oncogene. 2022;41:4769–78. 36182970 10.1038/s41388-022-02479-4 PMC10591256

[5] Mawe GM, Hoffman JM. Serotonin signalling in the gut-functions, dysfunctions and therapeutic targets. Nat Rev Gastroenterol Hepatol. 2013;10:473–86. 23797870 10.1038/nrgastro.2013.105 PMC4048923

[6] Kannen V, Bader M, Sakita JY, Uyemura SA, Squire JA. The dual role of Serotonin in Colorectal cancer. Trends Endocrinol Metab. 2020;31:611–25. 32439105 10.1016/j.tem.2020.04.008

[7] Huang, Wang Y, Thompson JW, Yin T, Alexander PB, Qin D, et al. Cancer-cell-derived GABA promotes β-catenin-mediated tumour growth and immunosuppression. Nat Cell Biol. 2022;24:230–41. 35145222 10.1038/s41556-021-00820-9 PMC8852304

[8] Kleinrok Z, Matuszek M, Jesipowicz J, Matuszek B, Opolski A, Radzikowski C. GABA content and GAD activity in colon tumors taken from patients with colon cancer or from xenografted human colon cancer cells growing as s.c. tumors in athymic nu/nu mice. J Physiol Pharm. 1998;49:303–10. 9670113

[9] Zhang B, Vogelzang A, Miyajima M, Sugiura Y, Wu Y, Chamoto K, et al. B cell-derived GABA elicits IL-10. Nature. 2021;599:471–6. 34732892 10.1038/s41586-021-04082-1 PMC8599023

[10] Huang D, Alexander PB, Li Q-J, Wang X-F. GABAergic signaling beyond synapses: an emerging target for cancer therapy. Trends Cell Biol. 2023;33:403–12. 36114091 10.1016/j.tcb.2022.08.004 PMC10008753

[11] Wang H, Zhang H, Sun Z, Chen W, Miao C. GABAB receptor inhibits tumor progression and epithelial-mesenchymal transition via the regulation of Hippo/YAP1 pathway in colorectal cancer. Int J Biol Sci. 2021;17:1953–62. 34131398 10.7150/ijbs.58135 PMC8193267

[12] Shu Q, Liu J, Liu X, Zhao S, Li H, Tan Y, et al. GABAB R/GSK-3β/NF-κB signaling pathway regulates the proliferation of colorectal cancer cells. Cancer Med. 2016;5:1259–67. 27060477 10.1002/cam4.686 PMC4924384

[13] Park SE, Georgescu A, Huh D. Organoids-on-a-chip. Science. 2019;364:960–5. 31171693 10.1126/science.aaw7894 PMC7764943

[14] Shirure VS, Hughes CCW, George SC. Engineering vascularized Organoid-on-a-Chip models. Annu Rev Biomed Eng. 2021;23:141–67. 33756087 10.1146/annurev-bioeng-090120-094330

[15] Tan SY, Feng X, Cheng LKW, Wu AR. Vascularized human brain organoid on-chip. Lab Chip. 2023;23:2693–709. 37256563 10.1039/d2lc01109c

[16] Achberger K, Probst C, Haderspeck J, Bolz S, Rogal J, Chuchuy J, et al. Merging organoid and organ-on-a-chip technology to generate complex multi-layer tissue models in a human retina-on-a-chip platform. Elife. 2019;8. 10.7554/eLife.46188 PMC6777939 31451149

[17] Hassell BA, Goyal G, Lee E, Sontheimer-Phelps A, Levy O, Chen CS, et al. Human organ chip models recapitulate orthotopic lung cancer growth, therapeutic responses, and tumor dormancy in vitro. Cell Rep. 2018;23:3698. 29925009 10.1016/j.celrep.2018.06.028

[18] Hiratsuka K, Miyoshi T, Kroll KT, Gupta NR, Valerius MT, Ferrante T, et al. Organoid-on-a-chip model of human ARPKD reveals mechanosensing pathomechanisms for drug discovery. Sci Adv. 2022;8:eabq0866. 36129975 10.1126/sciadv.abq0866 PMC9491724

[19] Zhang J, Tavakoli H, Ma L, Li X, Han L, Li X. Immunotherapy discovery on tumor organoid-on-a-chip platforms that recapitulate the tumor microenvironment. Adv Drug Deliv Rev. 2022;187:114365. 35667465 10.1016/j.addr.2022.114365

[20] Gunti S, Hoke ATK, Vu KP, London NR, Jr. Organoid and spheroid tumor models: techniques and applications. Cancers. 2021;13:874. 10.3390/cancers13040874 PMC7922036 33669619

[21] Shirure VS, Bi Y, Curtis MB, Lezia A, Goedegebuure MM, Goedegebuure SP, et al. Tumor-on-a-chip platform to investigate progression and drug sensitivity in cell lines and patient-derived organoids. Lab Chip. 2018;18:3687–702. 30393802 10.1039/c8lc00596f PMC10644986

[22] Aleman J, Skardal A. A multi-site metastasis-on-a-chip microphysiological system for assessing metastatic preference of cancer cells. Biotechnol Bioeng. 2019;116:936–44. 30450540 10.1002/bit.26871 PMC6399040

[23] Gil JF, Moura CS, Silverio V, Goncalves G, Santos HA. Cancer models on chip: paving the way to large-scale trial applications. Adv Mater. 2023;35:e2300692. 37103886 10.1002/adma.202300692

[24] Zhou Z, Cong L, Cong X. Patient-derived organoids in precision medicine: drug screening, Organoid-on-a-Chip and living organoid biobank. Front Oncol. 2021;11:762184. 35036354 10.3389/fonc.2021.762184 PMC8755639

[25] Vlachogiannis G, Hedayat S, Vatsiou A, Jamin Y, Fernandez-Mateos J, Khan K, et al. Patient-derived organoids model treatment response of metastatic gastrointestinal cancers. Science. 2018;359:920–6. 29472484 10.1126/science.aao2774 PMC6112415

[26] van de Wetering M, Francies HE, Francis JM, Bounova G, Iorio F, Pronk A, et al. Prospective derivation of a living organoid biobank of colorectal cancer patients. Cell. 2015;161:933–45. 25957691 10.1016/j.cell.2015.03.053 PMC6428276

[27] Lorenzo-Mart¡n LF, Broguiere N, Langer J, Tillard L, Nikolaev M, Coukos G, et al. Patient-derived mini-colons enable long-term modeling of tumor-microenvironment complexity. Nat Biotechnol. 2025;43:727–36. 38956326 10.1038/s41587-024-02301-4

[28] Sato T, Stange DE, Ferrante M, Vries RG, Van Es JH, Van den Brink S. et al. Long-term expansion of epithelial organoids from human colon, adenoma, adenocarcinoma, and Barrett's epithelium. Gastroenterology. 2011;141:1762–72. 21889923 10.1053/j.gastro.2011.07.050

[29] Sato T, Vries RG, Snippert HJ, van de Wetering M, Barker N, Stange DE, et al. Single Lgr5 stem cells build crypt-villus structures in vitro without a mesenchymal niche. Nature. 2009;459:262–5. 19329995 10.1038/nature07935

[30] Kim S, Choung S, Sun RX, Ung N, Hashemi N, Fong EJ, et al. Comparison of Cell and Organoid-Level Analysis of Patient-Derived 3D Organoids to Evaluate Tumor Cell Growth Dynamics and Drug Response. SLAS Discov. 2020;25:744–54. 32349587 10.1177/2472555220915827 PMC7372585

[31] Strelez C, Chilakala S, Ghaffarian K, Lau R, Spiller E, Ung N, et al. Human colorectal cancer-on-chip model to study the microenvironmental influence on early metastatic spread. iScience. 2021;24:102509. 34113836 10.1016/j.isci.2021.102509 PMC8169959

[32] Strelez C, Ghaffarian K, Mumenthaler SM. Multiplexed imaging and effluent analysis to monitor cancer cell intravasation using a colorectal cancer-on-chip. STAR Protoc. 2021;2:100984. 34927093 10.1016/j.xpro.2021.100984 PMC8649947

[33] Kimura R, Kasamatsu A, Koyama T, Fukumoto C, Kouzu Y, Higo M, et al. Glutamate acid decarboxylase 1 promotes metastasis of human oral cancer by β-catenin translocation and MMP7 activation. BMC Cancer. 2013;13:555. 24261884 10.1186/1471-2407-13-555 PMC3866561

[34] Marisa L, de Reyniès A, Duval A, Selves J, Gaub MP, Vescovo L, et al. Gene expression classification of colon cancer into molecular subtypes: characterization, validation, and prognostic value. PLoS Med. 2013;10:e1001453. 23700391 10.1371/journal.pmed.1001453 PMC3660251

[35] Venook AP, Niedzwiecki D, Lenz HJ, Innocenti F, Fruth B, Meyerhardt JA, et al. Effect of first-line chemotherapy combined with Cetuximab or Bevacizumab on overall survival in patients with KRAS wild-type advanced or metastatic colorectal cancer: a randomized clinical trial. JAMA. 2017;317:2392–401. 28632865 10.1001/jama.2017.7105 PMC5545896

[36] Kasendra M, Luc R, Yin J, Manatakis DV, Kulkarni G, Lucchesi C. Duodenum Intestine-Chip for preclinical drug assessment in a human relevant model. Elife. 2020;9:e50135 31933478 10.7554/eLife.50135 PMC6959988

[37] Basson MD. Paradigms for mechanical signal transduction in the intestinal epithelium. Category: Mol, cell Dev Biol Digest. 2003;68:217–25. 10.1159/000076385 14739529

[38] Godlewski J, Kmiec Z. Colorectal cancer invasion and atrophy of the enteric nervous system: potential feedback and impact on cancer progression. Int J Mol Sci. 2020;21(9):3391 32403316 10.3390/ijms21093391 PMC7247003

[39] Yan H, Tang G, Wang H, Hao L, He T, Sun X, et al. DNA methylation reactivates GAD1 expression in cancer by preventing CTCF-mediated polycomb repressive complex 2 recruitment. Oncogene. 2016;35:3995–4008. 26549033 10.1038/onc.2015.423

[40] Niu G, Deng L, Zhang X, Hu Z, Han S, Xu K, et al. GABRD promotes progression and predicts poor prognosis in colorectal cancer. Open Med. 2020;15:1172–83. 10.1515/med-2020-0128 PMC7718617 33336074

[41] Jin X, Demere Z, Nair K, Ali A, Ferraro GB, Natoli T, et al. A metastasis map of human cancer cell lines. Nature. 2020;588:331–6. 33299191 10.1038/s41586-020-2969-2 PMC8439149

[42] Neman J, Termini J, Wilczynski S, Vaidehi N, Choy C, Kowolik CM, et al. Human breast cancer metastases to the brain display GABAergic properties in the neural niche. Proc Natl Acad Sci USA. 2014;111:984–9. 24395782 10.1073/pnas.1322098111 PMC3903266

[43] Martirosian V, Deshpande K, Zhou H, Shen K, Smith K, Northcott P, et al. Medulloblastoma uses GABA transaminase to survive in the cerebrospinal fluid microenvironment and promote leptomeningeal dissemination. Cell Rep. 2021;35:109302. 34192534 10.1016/j.celrep.2021.109302 PMC8848833

[44] Schnepp PM, Lee DD, Guldner IH, O’Tighearnaigh TK, Howe EN, Palakurthi B, et al. GAD1 upregulation programs aggressive features of cancer cell metabolism in the brain metastatic microenvironment. Cancer Res. 2017;77:2844–56. 28400476 10.1158/0008-5472.CAN-16-2289 PMC5461057

[45] Skardal A, Devarasetty M, Forsythe S, Atala A, Soker S. A reductionist metastasis-on-a-chip platform for in vitro tumor progression modeling and drug screening. Biotechnol Bioeng. 2016;113:2020–32. 26888480 10.1002/bit.25950 PMC5778914

[46] Chen MB, Hajal C, Benjamin DC, Yu C, Azizgolshani H, Hynes RO, et al. Inflamed neutrophils sequestered at entrapped tumor cells via chemotactic confinement promote tumor cell extravasation. Proc Natl Acad Sci USA. 2018;115:7022–7. 29915060 10.1073/pnas.1715932115 PMC6142213

[47] Ayuso JM, Rehman S, Virumbrales-Munoz M, McMinn PH, Geiger P, Fitzgerald C, et al. Microfluidic tumor-on-a-chip model to evaluate the role of tumor environmental stress on NK cell exhaustion. Sci Adv.2021;7:eabc2331 33597234 10.1126/sciadv.abc2331 PMC7888951

[48] Modest DP, Ricard I, Heinemann V, Hegewisch-Becker S, Schmiegel W, Porschen R, et al. Outcome according to KRAS-, NRAS- and BRAF-mutation as well as KRAS mutation variants: pooled analysis of five randomized trials in metastatic colorectal cancer by the AIO colorectal cancer study group. Ann Oncol. 2016;27:1746–53. 27358379 10.1093/annonc/mdw261 PMC4999563

[49] Ozer M, Goksu SY, Sanford NN, Ahn C, Beg MS, Ali Kazmi SM. Age-dependent prognostic value of KRAS mutation in metastatic colorectal cancer. Future Oncol. 2021;17:4883–93. 34758634 10.2217/fon-2021-0650 PMC8890131

[50] Tsilimigras DI, Ntanasis-Stathopoulos I, Bagante F, Moris D, Cloyd J, Spartalis E, et al. Clinical significance and prognostic relevance of KRAS, BRAF, PI3K and TP53 genetic mutation analysis for resectable and unresectable colorectal liver metastases: A systematic review of the current evidence. Surg Oncol. 2018;27:280–8. 29937183 10.1016/j.suronc.2018.05.012

[51] Knowles SJ, Stafford AM, Zaman T, Angara K, Williams MR, Newbern JM, et al. Distinct hyperactive RAS/MAPK alleles converge on common GABAergic interneuron core programs. Development. 2023;150(10):dev201371 37254876 10.1242/dev.201371 PMC10281549

[52] Ryu HH, Kang M, Hwang KD, Jang HB, Kim SJ, Lee YS. Neuron type-specific expression of a mutant KRAS impairs hippocampal-dependent learning and memory. Sci Rep. 2020;10:17730. 33082413 10.1038/s41598-020-74610-y PMC7575532

[53] Duchalais E, Guilluy C, Nedellec S, Touvron M, Bessard A, Touchefeu Y, et al. Colorectal cancer cells adhere to and migrate along the neurons of the enteric nervous system. Cell Mol Gastroenterol Hepatol. 2018;5:31–49. 29188232 10.1016/j.jcmgh.2017.10.002 PMC5696385

