# Supplementary material for: GABAergic signaling contributes to tumor cell invasion and poor overall survival in colorectal cancer
Source: Oncogene. 2025 Aug 24;44(41):3924–38. doi: 10.1038/s41388-025-03546-2 (PMC12500471; doi:10.1038/s41388-025-03546-2)
Supplement: Supplementary file 1 — Supplemental Material Combined [file 41388_2025_3546_MOESM1_ESM.docx]

Supplementary Materials for

**GABAergic signaling contributes to tumor cell invasion and poor overall survival in colorectal cancer**

Carly Strelez *et al.*

*Corresponding author Shannon Mumenthaler. Email: [smumenthaler@emila.org](mailto:smumenthaler@emila.org)

**This PDF file includes:**

Figs. S1 to S7

Tables S1 to S4

Supplementary Figures.


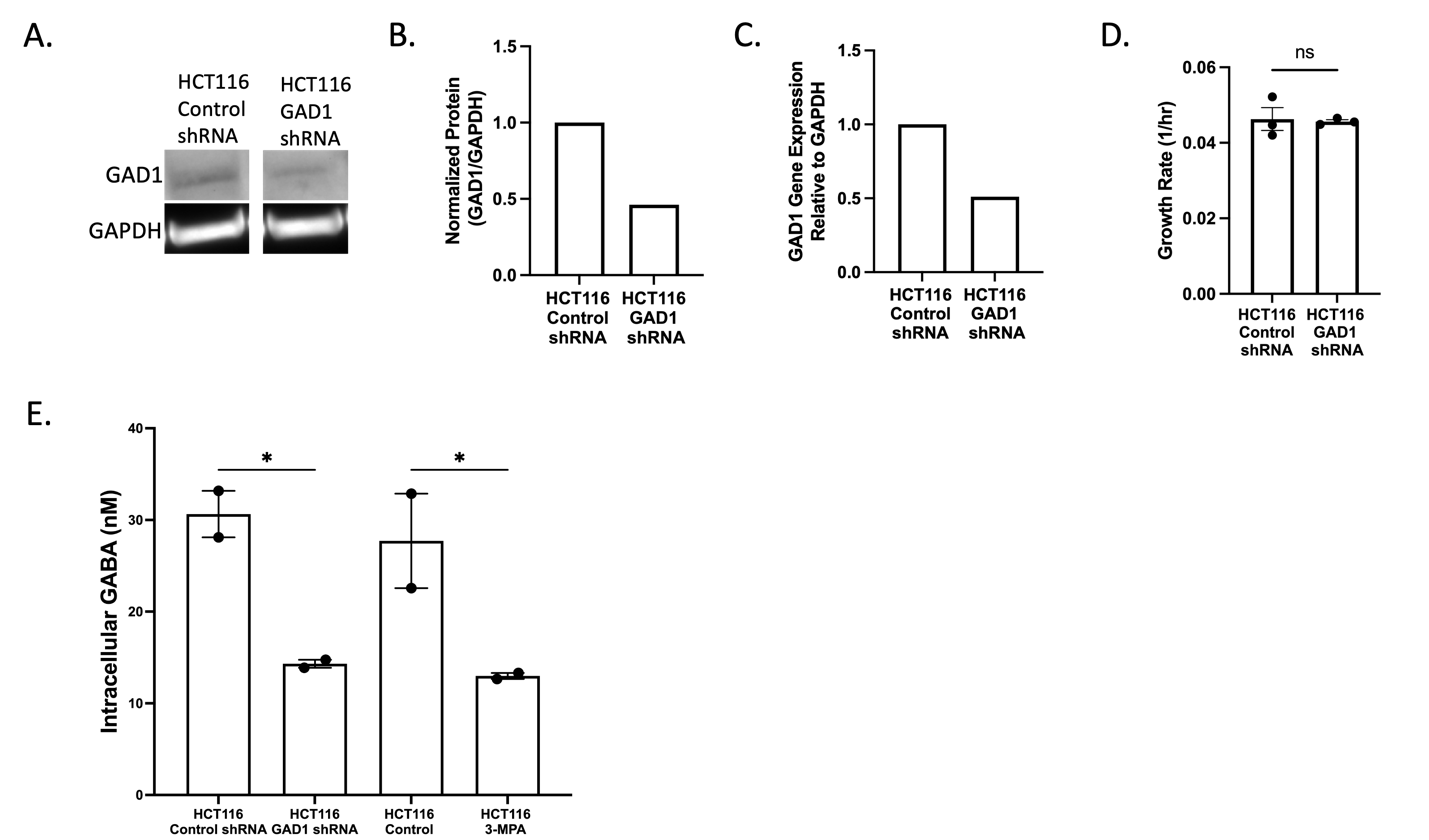


**Supplemental Figure 1. *GAD1* knockdown in HCT116 tumor cell.** (A.) Cropped western blot and (B.) quantification confirm GAD1 knockdown in HCT116 tumor cells. (C.) *GAD1* gene expression in HCT116 control shRNA cells or HCT116 GAD1 shRNA cells. (D.) Growth rate of *GAD1*-knockdown or control HCT116 tumor cells when grown in traditional cell culture methods. N=3. Individual data are shown and mean ± SEM are represented. Data was analyzed using a t-test; ns=not significant. (E.) GABA in cell lysates confirm GAD1 inhibition in GAD1 shRNA or 3-MPA-treated HCT116 tumor cells. N=2 technical replicates. Individual data are shown and mean ± SEM are represented. Data was analyzed using a one-way ANOVA with Šídák’s multiple comparison test.

**
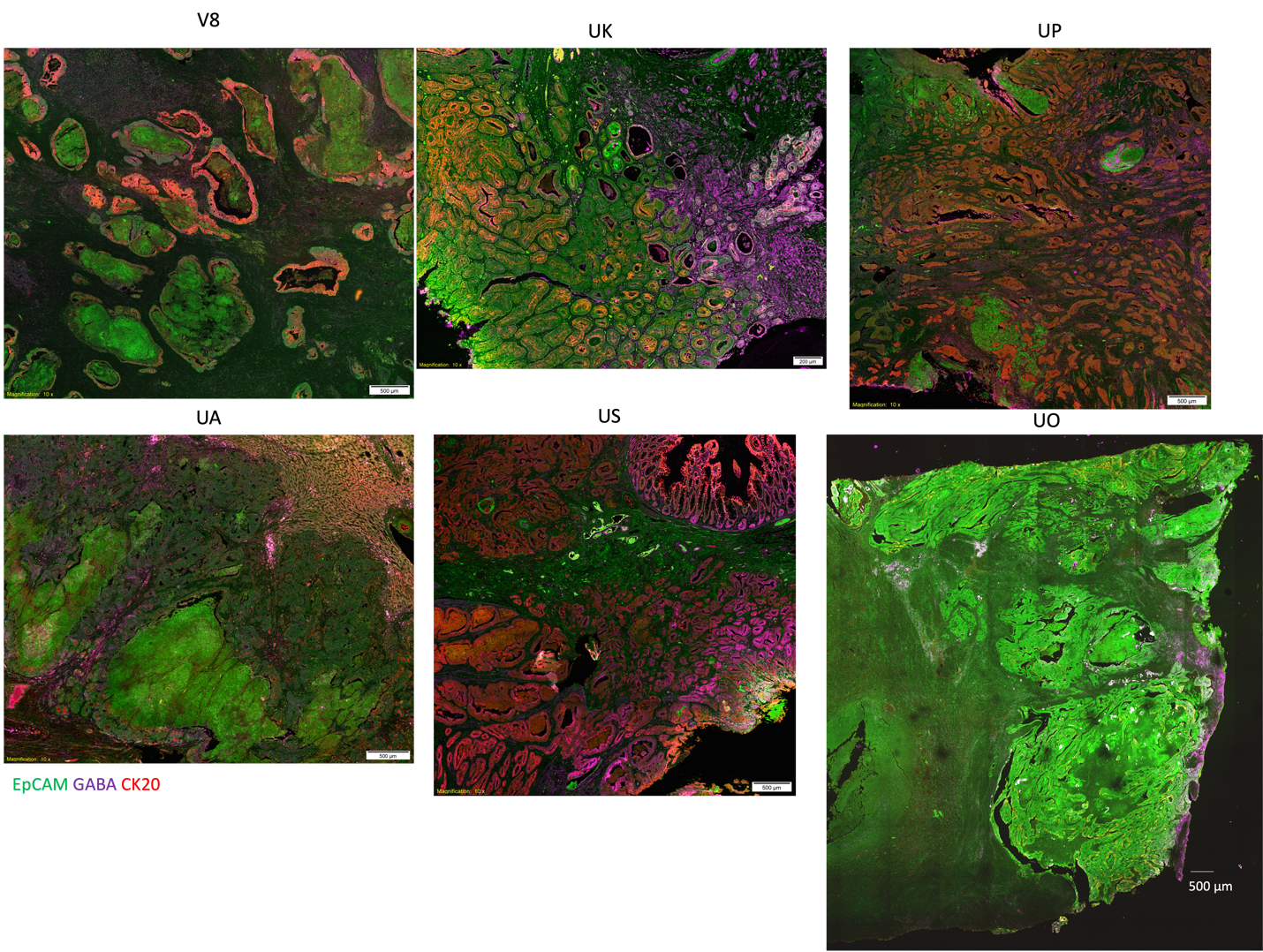
 Supplemental Figure 2. GABA in CRC Tumors.** Un-cropped representative images of 10x immunofluorescence images of the 5 tumors stained for EpCam (green), CK20 (red), and GABA (purple). Scale bars represent 500 μm and 200 μm for UK.


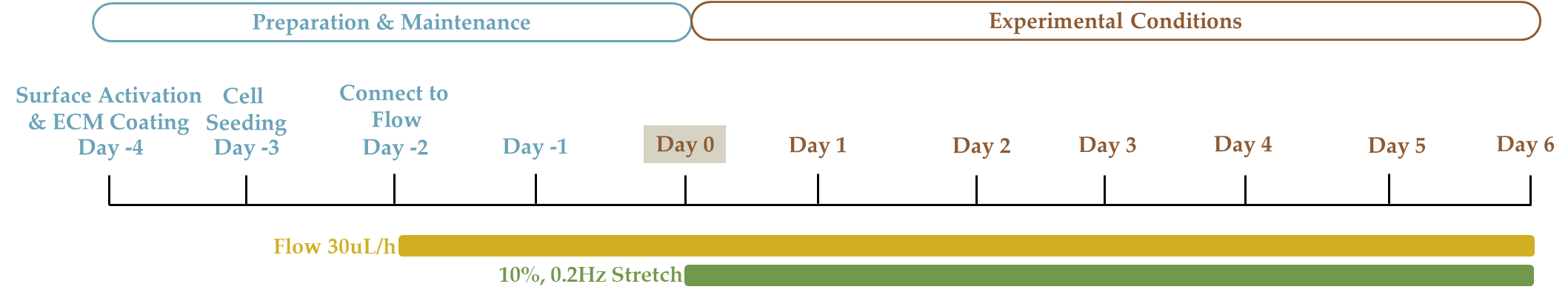


Supplemental Figure 3. Experimental Timeline. Detailed overview of key steps on critical days of organoid-on-chip preparation and experimental conditions.


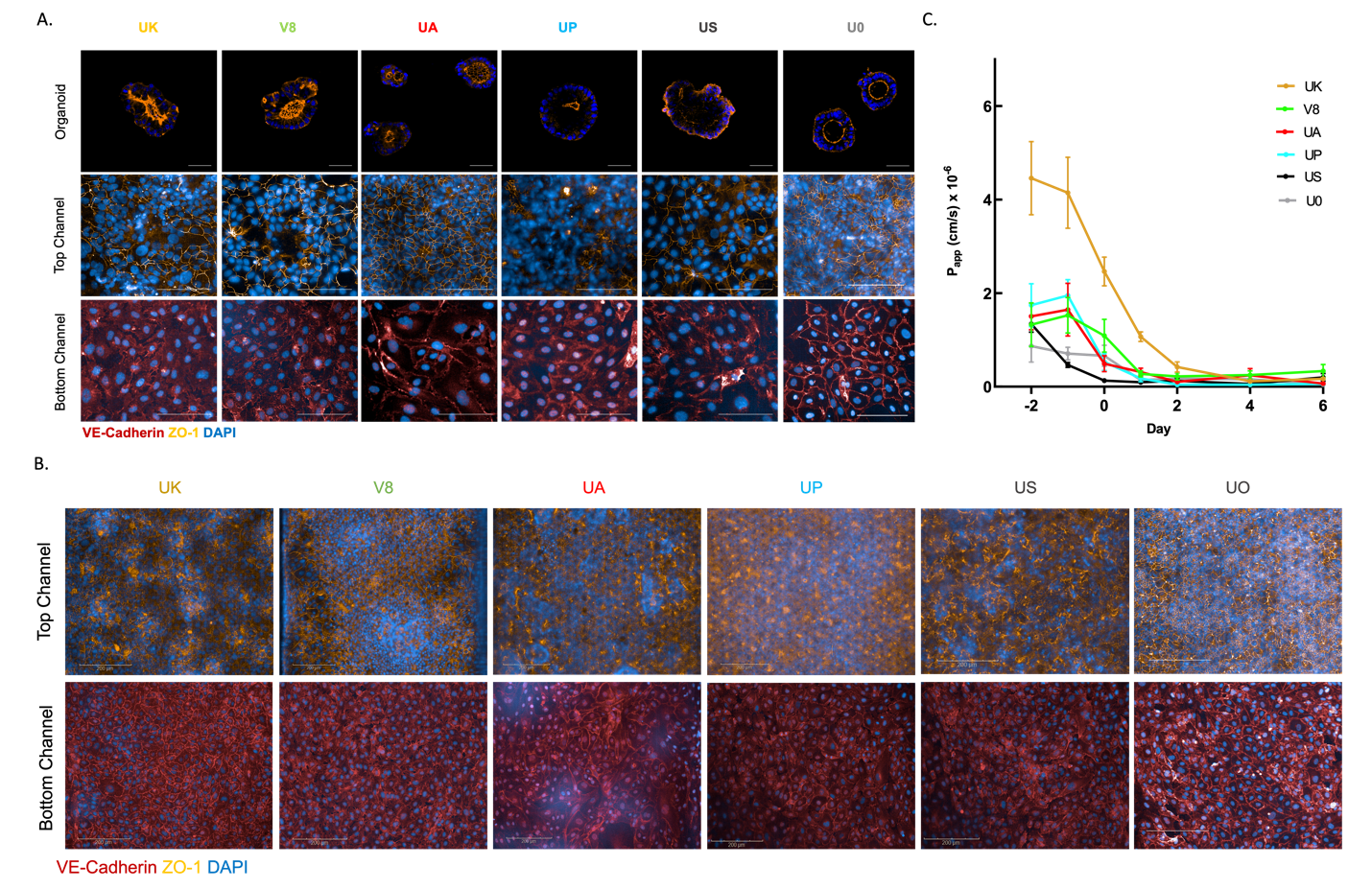


**Supplemental Figure 4**. **Tight junction formation in the organoid-on-chip. (A).** 20x confocal fluorescent images of ZO-1 (gold) and VE-Cadherin (red) staining of the organoid-chips.  **(B.)** Large scale confocal fluorescent images of the epithelial (top panel) and endothelial (bottom panel) channels of the organoid-chips on day 6 stained for tight junction protein ZO-1 (gold) and VE-Cadherin (red). Cell nuclei are labeled with DAPI (blue). Scale bars represent 200 μm. **(C.)** Apparent permeability (P_app_) of the organoid chips over the 6 day experiment.


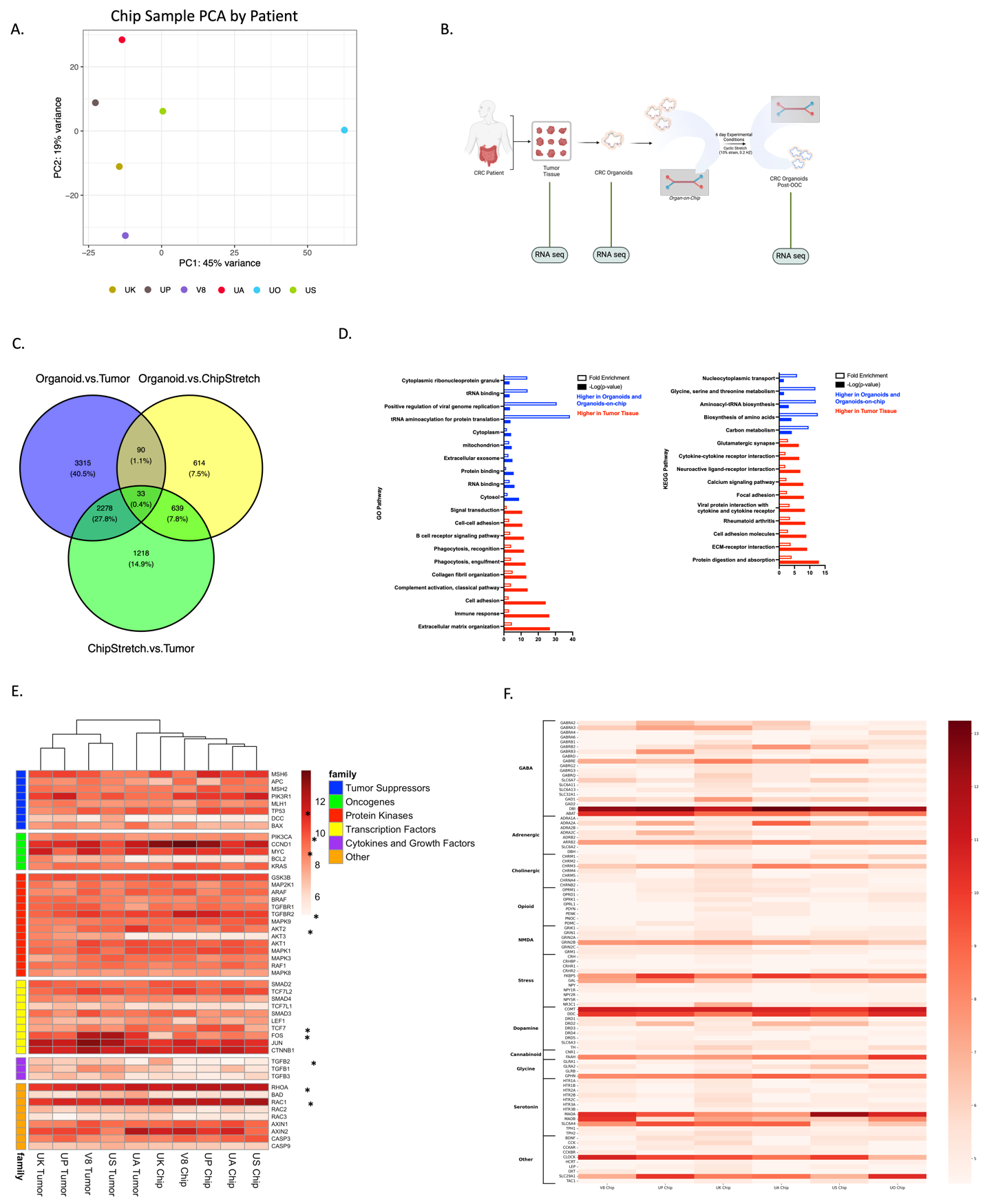


**Supplemental Figure 5. Model validation using gene expression analysis.** **(A.)** Principal component analysis (PCA) of the RNA-seq of the CRC organoid-on-chips demonstrates the patient heterogeneity represented in the genes. Each dot represents one replicate of stretched, CRC organoid-on-chips and each dot is colored by donor. **(B.)** RNAseq was performed on the patient-matched tumor tissue, patient-derived organoid in standard 3-D culture, and after completion of organoid-chip experiments. **(C.)** Differential gene expression analysis was carried out to identify genes that are up or down-regulated (*P*<0.05) in the CRC organoid-on-chip compared to the organoids alone (yellow), CRC organoid-on-chip with fluid flow and rhythmic stretching compared to tumor (green), or the organoid compared to the tumor (blue). The numbers of unique and overlapping genes were identified and represented in a Venn Diagram. N = 5 independent donors and organoid, organoid-on-chip, and tumor tissue were patient-matched. **(D.)** Genes that were upregulated in organoids and organoid-chips as compared to the tumor tissue (blue) and genes that were upregulated in the tumor tissue as compared to organoids and organoid-chips (red) were subjected to over-enrichment analysis using DAVID software. **(E.)** Heatmap of expression of key genes related to colorectal cancer progression compared between the patient-matched tumor and organoid-chips. Statistically significant genes (*P_adj_* < 0.05) from a differentially expressed gene analysis comparing the organoid-chips and the tumor tissue are highlighted with asterisks. (**F.**) Expression heatmap of neurotransmitter-gene sets compared between the organoid-chips.


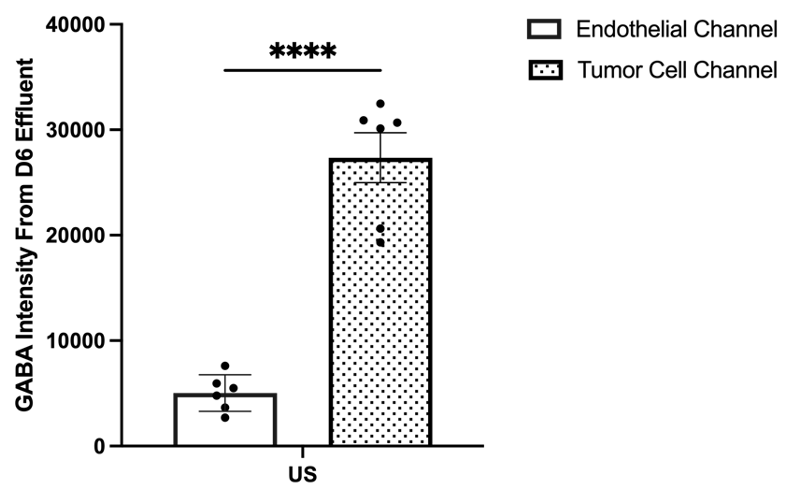


**Supplemental Figure 6.** GABA levels in tumor cell channel and endothelial cell channel in US organoid-chips, measured on day 6. N=6 chips. Individual raw data are shown and mean ± SEM are represented. Data was analyzed using an unpaired t-test; *****P* < 0.0001.


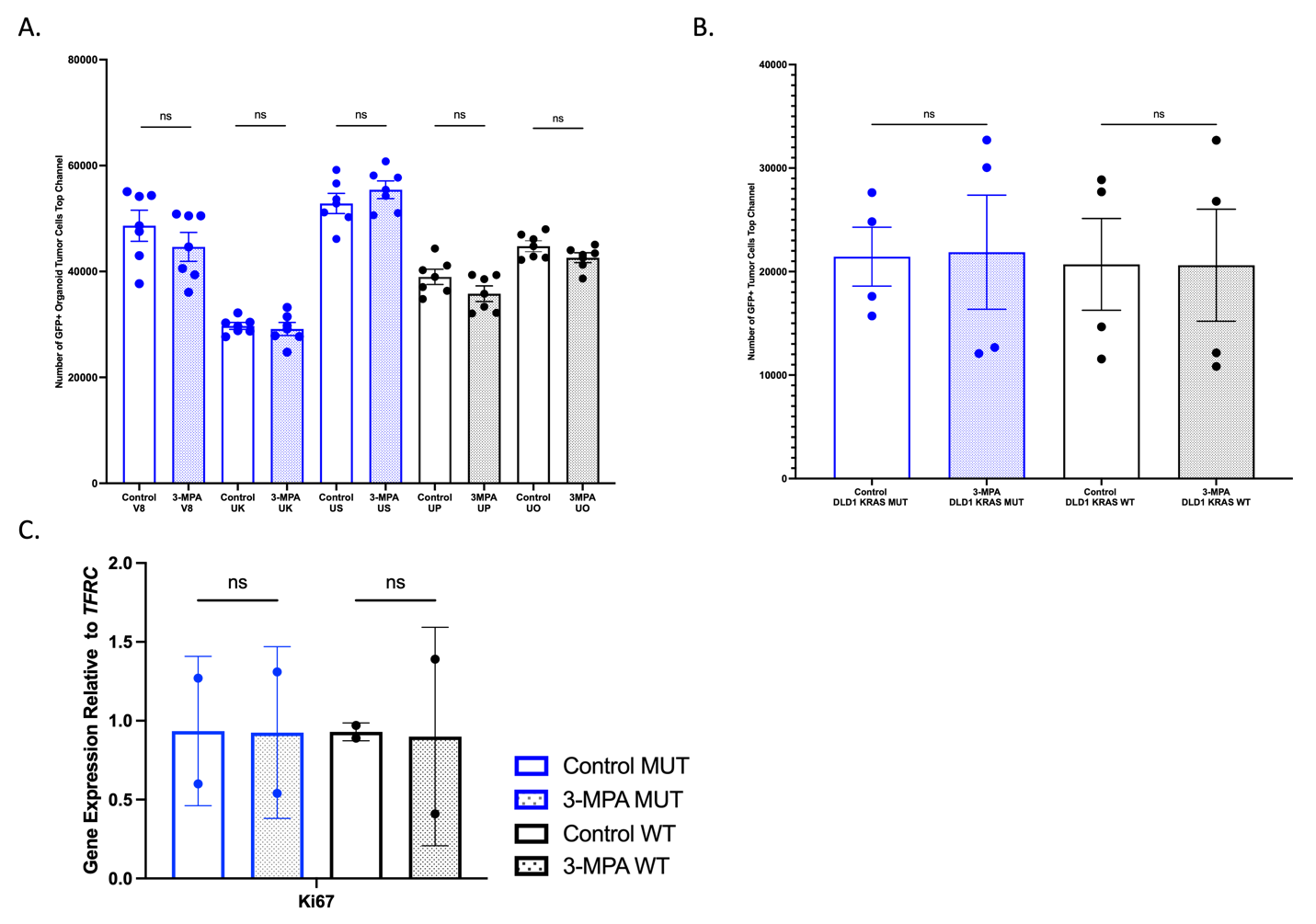


**Supplemental Figure 7. Tumor cell counts with and without 3-MPA.** **(A.)** Numbers of organoid tumor cells present in the top channel on D6 with or without 3-MPA treatment. N=6 chips; ns = not significant. Data was analyzed using an unpaired t-test. All data are shown as mean ± SEM. **(B.)** Numbers of DLD1 isogenic *KRAS* tumor cells present in the top channel on D6 with or without 3-MPA treatment. N=4 chips; ns = not significant. Data was analyzed using a one-way ANOVA with Šídák’s multiple comparison test. All data are shown as mean ± SEM. **(C.)** *Ki67* gene expression of DLD1 isogenic *KRAS* cell lines with or without 3-MPA treatment. Cells from the top channel of 5 chips across two biological replicates were harvested for qPCR. Circles on the graph represent the mean of 3 technical replicates. N=2 biological replicates for statistical analysis; ns = not significant. Data was analyzed using a one-way ANOVA with Šídák’s multiple comparison test. All data are shown as mean ± SEM.

**Supplementary Tables.**

**Table S1. Patient and tumor characteristics of the CALGB/SWOG 80405 cohort.**

| **Table 1. Patient Characteristics** | | | | |
| --- | --- | --- | --- | --- |
|  | **BEV Arm (N = 226)** | **CET Arm (N = 207)** | **Total (N = 433)** | ***P*-Value^*^** |
| **Age (yrs)** | | |  |  |
| **Median (range)** | 60 (24, 80) | 59 (30, 83) | 60 (24, 83) | 0.94 |
| **Gender** | | |  | 0.37 |
| **Male** | 146 (65 %) | 125 (60 %) | 271 (63 %) |  |
| **Female** | 80 (35 %) | 82 (40 %) | 162 (37 %) |  |
| **Performance Score** | | | | 1.00 |
| **0** | 131 (58 %) | 120 (58 %) | 251 (58 %) |  |
| **1** | 95 (42 %) | 87 (42 %) | 182 (42 %) |  |
| **Number of Metastases** | | |  | 0.28 |
| **1** | 118 (52 %) | 104 (50 %) | 222 (51 %) |  |
| **2** | 69 (31 %) | 78 (38 %) | 147 (34 %) |  |
| **3+** | 37 (16 %) | 24 (12 %) | 61 (14 %) |  |
| **Unknown** | 2 (1 %) | 1 (< 1 %) | 3 (1 %) |  |
| **Planned Protocol Chemotherapy** | | |  | 0.27 |
| **FOLFOX** | 174 (77 %) | 149 (72 %) | 323 (75 %) |  |
| **FOLFIRI** | 52 (23 %) | 58 (28 %) | 110 (25 %) |  |
| **Tumor Location** | | |  | 0.41 |
| **Left** | 128 (57 %) | 130 (63 %) | 258 (60 %) |  |
| **Right/Transverse** | 95 (42 %) | 74 (36 %) | 169 (39 %) |  |
| **Unknown** | 3 (1 %) | 3 (1 %) | 6 (1 %) |  |
| **K*RAS* Mutation** | | | | 0.51 |
| **Wild-type** | 193 (85 %) | 172 (83 %) | 365 (84 %) |  |
| **Mutant** | 32 (14 %) | 35 (17 %) | 67 (15 %) |  |
| **Unknown** | 1 (< 1 %) | 0 (0 %) | 1 (< 1 %) |  |
| ***BRAF* Mutation** | | |  | **0.042** |
| **Wild-type** | 175 (77 %) | 159 (77 %) | 334 (77 %) |  |
| **Mutant** | 37 (16 %) | 23 (11 %) | 60 (14 %) |  |
| **Unknown** | 14 (6 %) | 25 (12 %) | 39 (9 %) |  |
| **MSI Status from PCR** | | |  | **0.027** |
| **MSI-H** | 18 (8 %) | 11 (5 %) | 29 (7 %) |  |
| **MSI-L** | 9 (4 %) | 10 (5 %) | 19 (4 %) |  |
| **MSS** | 181 (80 %) | 151 (73 %) | 332 (77 %) |  |
| **Unknown** | 18 (8 %) | 35 (17 %) | 53 (12 %) |  |

**P*-value from Wilcoxon rank-sum test for continuous variables; Fisher’s exact test for categorical variables

**Table S2. Association between GABA pathway genes expression and outcomes in GALGB/SWOG 80405 patients (overall cohort).**

|  |  |  | PFS | | | | | |  | OS | | | | | |
| --- | --- | --- | --- | --- | --- | --- | --- | --- | --- | --- | --- | --- | --- | --- | --- |
|  |  |  |  | | Univariable Analysis | | Multivariable Analysis | |  |  | | Univariable Analysis | | Multivariable Analysis | |
| Gene | Group |  | Events/ Patients | Median Survival (95% CI) | HR (95% CI) | *P^*^* | HR (95% CI) | *P^*^* |  | Events/ Patients | Median Survival (95% CI) | HR (95% CI) | *P^*^* | HR (95% CI) | *P^*^* |
| GABBR1 (scale: 0.75) | continuous |  | 405/433 | 11.1 (10.1, 12.1) | 1.05 (0.95, 1.17) | 0.3 | 0.98 (0.88, 1.1) | 0.78 |  | 360/433 | 30.6 (27.7, 34.1) | 1.06 (0.96, 1.18) | 0.25 | 0.96 (0.85, 1.08) | 0.51 |
| GABBR1 | low |  | 139/145 | 11.2 (9.5, 14.0) | Reference | **0.036** | Reference | 0.22 |  | 120/145 | 32.8 (26.9, 37.5) | Reference | 0.26 | Reference | 0.97 |
|  | mid |  | 130/144 | 12.5 (11.0, 15.2) | 0.89 (0.70, 1.14) |  | 0.83 (0.64, 1.09) |  |  | 117/144 | 34.1 (29.0, 38.6) | 1.03 (0.80, 1.33) |  | 0.97 (0.74, 1.27) |  |
|  | high |  | 136/144 | 10.1 (9.2, 11.5) | 1.22 (0.96, 1.55) |  | 1.02 (0.79, 1.33) |  |  | 123/144 | 25.9 (23.5, 32.5) | 1.21 (0.94, 1.56) |  | 0.97 (0.74, 1.29) |  |
| GABBR2 (scale: 1.75) | continuous |  | 405/433 | 11.1 (10.1, 12.1) | 0.94 (0.85, 1.05) | 0.27 | 0.95 (0.85, 1.06) | 0.35 |  | 360/433 | 30.6 (27.7, 34.1) | 0.96 (0.86, 1.07) | 0.44 | 0.98 (0.87, 1.09) | 0.69 |
| GABBR2 | low |  | 139/145 | 11.2 (10.1, 13.5) | Reference | 0.46 | Reference | 0.55 |  | 127/145 | 30.9 (26.3, 35.8) | Reference | 0.53 | Reference | 0.62 |
|  | mid |  | 135/144 | 11.1 (9.3, 12.9) | 0.92 (0.72, 1.16) |  | 0.94 (0.73, 1.23) |  |  | 118/144 | 30.2 (26.7, 34.3) | 1.01 (0.78, 1.29) |  | 1.07 (0.82, 1.41) |  |
|  | high |  | 131/144 | 11.0 (9.3, 13.0) | 0.86 (0.68, 1.09) |  | 0.87 (0.67, 1.12) |  |  | 115/144 | 31.2 (25.0, 36.9) | 0.88 (0.69, 1.14) |  | 0.94 (0.72, 1.23) |  |
| GABRA1 (scale: 1.62) | continuous |  | 405/433 | 11.1 (10.1, 12.1) | 0.97 (0.88, 1.08) | 0.59 | 0.94 (0.84, 1.05) | 0.26 |  | 360/433 | 30.6 (27.7, 34.1) | 0.97 (0.87, 1.08) | 0.53 | 0.92 (0.81, 1.04) | 0.18 |
| GABRA1 | low |  | 134/146 | 11.5 (10.2, 13.5) | Reference | 0.39 | Reference | 0.33 |  | 119/146 | 31.2 (27.7, 36.0) | Reference | 0.11 | Reference | **0.0089** |
|  | mid |  | 137/146 | 11.1 (9.2, 12.7) | 1.17 (0.92, 1.49) |  | 1.15 (0.88, 1.51) |  |  | 128/146 | 28.0 (22.4, 34.6) | 1.28 (1.00, 1.64) |  | 1.37 (1.04, 1.81) |  |
|  | high |  | 134/141 | 10.9 (9.2, 13.4) | 1.03 (0.81, 1.31) |  | 0.97 (0.72, 1.30) |  |  | 113/141 | 32.4 (26.3, 36.5) | 1.03 (0.80, 1.34) |  | 0.93 (0.67, 1.28) |  |
| GABRA2 (scale: 2.44) | continuous |  | 405/433 | 11.1 (10.1, 12.1) | 0.87 (0.79, 0.96) | **0.0066** | 0.94 (0.84, 1.04) | 0.21 |  | 360/433 | 30.6 (27.7, 34.1) | 0.86 (0.77, 0.95) | **0.005** | 0.91 (0.82, 1.02) | 0.1 |
| GABRA2 | low |  | 136/146 | 10.6 (9.2, 12.3) | Reference | 0.13 | Reference | 0.26 |  | 126/146 | 26.4 (22.4, 31.8) | Reference | 0.1 | Reference | 0.45 |
|  | mid |  | 135/145 | 10.3 (9.2, 11.7) | 0.94 (0.74, 1.19) |  | 1.14 (0.88, 1.47) |  |  | 118/145 | 33.3 (26.0, 38.8) | 0.83 (0.64, 1.07) |  | 1.03 (0.79, 1.34) |  |
|  | high |  | 134/142 | 12.9 (11.1, 15.9) | 0.79 (0.62, 1.00) |  | 0.93 (0.72, 1.20) |  |  | 116/142 | 33.8 (29.1, 37.4) | 0.77 (0.60, 0.99) |  | 0.87 (0.67, 1.14) |  |
| GABRA4 (scale: 2.7) | continuous |  | 405/433 | 11.1 (10.1, 12.1) | 0.92 (0.83, 1.01) | 0.074 | 0.95 (0.86, 1.05) | 0.34 |  | 360/433 | 30.6 (27.7, 34.1) | 0.93 (0.84, 1.04) | 0.2 | 0.98 (0.88, 1.09) | 0.71 |
| GABRA4 | low |  | 138/147 | 10.2 (9.2, 11.7) | Reference | 0.25 | Reference | 0.84 |  | 124/147 | 26.3 (23.5, 32.4) | Reference | 0.5 | Reference | 0.5 |
|  | mid |  | 133/142 | 11.0 (9.3, 12.9) | 0.90 (0.71, 1.14) |  | 0.95 (0.74, 1.21) |  |  | 118/142 | 33.6 (28.0, 36.7) | 0.88 (0.68, 1.13) |  | 0.87 (0.68, 1.13) |  |
|  | high |  | 134/144 | 12.7 (10.9, 14.9) | 0.82 (0.64, 1.04) |  | 0.93 (0.72, 1.19) |  |  | 118/144 | 33.4 (28.9, 36.0) | 0.88 (0.68, 1.13) |  | 1.00 (0.77, 1.30) |  |
| GABRA5 (scale: 1.53) | continuous |  | 405/433 | 11.1 (10.1, 12.1) | 1.07 (0.97, 1.18) | 0.18 | 1.02 (0.92, 1.14) | 0.7 |  | 360/433 | 30.6 (27.7, 34.1) | 1.07 (0.96, 1.19) | 0.22 | 1.01 (0.9, 1.14) | 0.83 |
| GABRA5 | low |  | 136/147 | 11.5 (10.1, 14.2) | Reference | 0.14 | Reference | 0.4 |  | 122/147 | 31.1 (26.1, 36.1) | Reference | 0.25 | Reference | 0.43 |
|  | mid |  | 134/143 | 11.7 (10.9, 13.3) | 1.08 (0.85, 1.37) |  | 1.01 (0.78, 1.31) |  |  | 116/143 | 33.9 (29.2, 37.4) | 0.99 (0.77, 1.27) |  | 0.92 (0.70, 1.22) |  |
|  | high |  | 135/143 | 9.8 (8.7, 11.4) | 1.26 (1.00, 1.60) |  | 1.17 (0.90, 1.54) |  |  | 122/143 | 26.4 (23.7, 33.4) | 1.19 (0.93, 1.54) |  | 1.10 (0.82, 1.46) |  |
| GABRA6 (scale: 1.63) | continuous |  | 405/433 | 11.1 (10.1, 12.1) | 0.92 (0.83, 1.03) | 0.13 | 0.85 (0.75, 0.97) | **0.013** |  | 360/433 | 30.6 (27.7, 34.1) | 0.98 (0.88, 1.09) | 0.71 | 0.9 (0.79, 1.03) | 0.11 |
| GABRA6 | low |  | 145/151 | 11.0 (10.0, 12.9) | Reference | 0.19 | Reference | **0.0098** |  | 129/151 | 30.9 (26.9, 34.7) | Reference | 0.57 | Reference | 0.091 |
|  | mid |  | 129/139 | 11.1 (9.2, 13.0) | 0.99 (0.78, 1.26) |  | 0.85 (0.65, 1.11) |  |  | 117/139 | 28.9 (23.5, 35.0) | 1.06 (0.83, 1.36) |  | 0.97 (0.73, 1.27) |  |
|  | high |  | 131/143 | 11.4 (9.8, 14.2) | 0.82 (0.65, 1.04) |  | 0.64 (0.47, 0.86) |  |  | 114/143 | 33.6 (26.5, 38.1) | 0.92 (0.72, 1.19) |  | 0.73 (0.52, 1.02) |  |
| GABRB1 (scale: 2.38) | continuous |  | 405/433 | 11.1 (10.1, 12.1) | 0.85 (0.77, 0.94) | **9.1e-04** | 0.88 (0.79, 0.97) | **0.013** |  | 360/433 | 30.6 (27.7, 34.1) | 0.88 (0.79, 0.98) | **0.015** | 0.95 (0.85, 1.06) | 0.37 |
| GABRB1 | low |  | 142/148 | 9.5 (8.4, 11.3) | Reference | **0.0032** | Reference | 0.073 |  | 126/148 | 26.1 (23.7, 32.6) | Reference | 0.11 | Reference | 0.79 |
|  | mid |  | 135/143 | 11.0 (9.5, 12.7) | 0.80 (0.63, 1.02) |  | 0.84 (0.66, 1.07) |  |  | 118/143 | 26.9 (24.2, 35.0) | 0.90 (0.70, 1.15) |  | 1.02 (0.79, 1.32) |  |
|  | high |  | 128/142 | 14.0 (11.3, 16.3) | 0.66 (0.52, 0.84) |  | 0.74 (0.58, 0.96) |  |  | 116/142 | 35.8 (31.3, 41.0) | 0.76 (0.59, 0.98) |  | 0.93 (0.71, 1.22) |  |
| GABRB3 (scale: 2.13) | continuous |  | 405/433 | 11.1 (10.1, 12.1) | 1.02 (0.93, 1.12) | 0.71 | 1.04 (0.94, 1.14) | 0.49 |  | 360/433 | 30.6 (27.7, 34.1) | 0.98 (0.89, 1.09) | 0.74 | 0.99 (0.89, 1.1) | 0.86 |
| GABRB3 | low |  | 141/147 | 11.7 (10.9, 14.2) | Reference | 0.93 | Reference | 0.97 |  | 122/147 | 31.3 (27.7, 35.8) | Reference | 1 | Reference | 0.52 |
|  | mid |  | 130/142 | 10.9 (9.8, 12.1) | 1.05 (0.82, 1.33) |  | 0.98 (0.76, 1.27) |  |  | 118/142 | 30.9 (25.2, 35.9) | 1.00 (0.78, 1.29) |  | 0.86 (0.65, 1.12) |  |
|  | high |  | 134/144 | 10.9 (9.2, 13.0) | 1.01 (0.79, 1.28) |  | 0.97 (0.75, 1.24) |  |  | 120/144 | 29.1 (25.6, 35.4) | 1.01 (0.78, 1.30) |  | 0.93 (0.71, 1.21) |  |
| GABRD (scale: 1.21) | continuous |  | 405/433 | 11.1 (10.1, 12.1) | 1.07 (0.97, 1.18) | 0.17 | 1.07 (0.96, 1.19) | 0.2 |  | 360/433 | 30.6 (27.7, 34.1) | 1.1 (0.99, 1.22) | 0.068 | 1.1 (0.98, 1.22) | 0.089 |
| GABRD | low |  | 135/145 | 12.1 (10.9, 16.1) | Reference | 0.15 | Reference | 0.05 |  | 114/145 | 33.7 (26.4, 39.1) | Reference | 0.13 | Reference | **0.036** |
|  | mid |  | 138/145 | 10.1 (9.2, 11.9) | 1.26 (0.99, 1.60) |  | 1.37 (1.06, 1.75) |  |  | 126/145 | 29.2 (25.9, 35.8) | 1.27 (0.99, 1.64) |  | 1.42 (1.08, 1.85) |  |
|  | high |  | 132/143 | 11.3 (9.6, 12.9) | 1.17 (0.92, 1.48) |  | 1.19 (0.93, 1.53) |  |  | 120/143 | 29.1 (25.3, 34.3) | 1.24 (0.96, 1.61) |  | 1.26 (0.96, 1.64) |  |
| GABRE (scale: 0.92) | continuous |  | 405/433 | 11.1 (10.1, 12.1) | 0.89 (0.81, 0.98) | **0.023** | 0.96 (0.86, 1.07) | 0.47 |  | 360/433 | 30.6 (27.7, 34.1) | 0.86 (0.77, 0.96) | **0.0075** | 0.89 (0.79, 1.01) | 0.078 |
| GABRE | low |  | 135/146 | 9.6 (7.8, 11.3) | Reference | 0.059 | Reference | 0.59 |  | 123/146 | 23.5 (17.9, 26.8) | Reference | 0.057 | Reference | 0.39 |
|  | mid |  | 133/145 | 11.0 (9.4, 12.7) | 0.80 (0.63, 1.02) |  | 0.89 (0.68, 1.15) |  |  | 120/145 | 32.6 (26.7, 35.9) | 0.76 (0.59, 0.98) |  | 0.83 (0.63, 1.09) |  |
|  | high |  | 137/142 | 13.1 (11.2, 16.1) | 0.76 (0.60, 0.97) |  | 0.88 (0.68, 1.15) |  |  | 117/142 | 34.4 (31.1, 38.7) | 0.77 (0.60, 0.99) |  | 0.86 (0.65, 1.14) |  |
| GABRG1 (scale: 1.71) | continuous |  | 405/433 | 11.1 (10.1, 12.1) | 1 (0.91, 1.1) | 0.97 | 0.95 (0.85, 1.07) | 0.41 |  | 360/433 | 30.6 (27.7, 34.1) | 1.04 (0.94, 1.15) | 0.45 | 0.98 (0.87, 1.11) | 0.75 |
| GABRG1 | low |  | 149/163 | 11.1 (10.1, 13.2) | Reference | 0.57 | Reference | 0.97 |  | 129/163 | 31.1 (26.4, 35.9) | Reference | 0.29 | Reference | 0.56 |
|  | mid |  | 124/129 | 11.5 (9.8, 14.4) | 1.10 (0.87, 1.40) |  | 0.99 (0.76, 1.29) |  |  | 113/129 | 31.2 (26.1, 36.8) | 1.19 (0.92, 1.53) |  | 1.14 (0.86, 1.52) |  |
|  | high |  | 132/141 | 10.3 (9.1, 11.7) | 1.13 (0.89, 1.43) |  | 0.97 (0.72, 1.29) |  |  | 118/141 | 29.1 (24.9, 35.1) | 1.19 (0.93, 1.53) |  | 1.02 (0.75, 1.38) |  |
| GABRG2 (scale: 2.21) | continuous |  | 405/433 | 11.1 (10.1, 12.1) | 0.96 (0.87, 1.06) | 0.45 | 0.92 (0.83, 1.03) | 0.14 |  | 360/433 | 30.6 (27.7, 34.1) | 0.99 (0.9, 1.1) | 0.89 | 0.94 (0.84, 1.06) | 0.3 |
| GABRG2 | low |  | 139/147 | 10.9 (9.2, 12.7) | Reference | 0.43 | Reference | 0.079 |  | 120/147 | 29.1 (26.1, 32.8) | Reference | 0.98 | Reference | 0.33 |
|  | mid |  | 132/142 | 11.0 (9.5, 13.0) | 0.88 (0.69, 1.12) |  | 0.79 (0.61, 1.02) |  |  | 120/142 | 29.9 (25.3, 36.7) | 1.02 (0.79, 1.31) |  | 0.87 (0.66, 1.15) |  |
|  | high |  | 134/144 | 11.7 (10.1, 14.1) | 0.87 (0.68, 1.10) |  | 0.76 (0.59, 0.99) |  |  | 120/144 | 33.9 (25.6, 36.8) | 0.99 (0.77, 1.28) |  | 0.81 (0.62, 1.07) |  |
| GABRG3 (scale: 2.1) | continuous |  | 405/433 | 11.1 (10.1, 12.1) | 0.96 (0.87, 1.06) | 0.39 | 0.94 (0.85, 1.05) | 0.26 |  | 360/433 | 30.6 (27.7, 34.1) | 0.97 (0.88, 1.07) | 0.56 | 0.96 (0.86, 1.07) | 0.45 |
| GABRG3 | low |  | 134/146 | 11.5 (10.2, 14.2) | Reference | **0.01** | Reference | **0.016** |  | 116/146 | 31.2 (28.9, 36.0) | Reference | **0.02** | Reference | 0.13 |
|  | mid |  | 145/150 | 9.6 (9.0, 11.4) | 1.33 (1.05, 1.69) |  | 1.35 (1.04, 1.76) |  |  | 132/150 | 26.3 (22.8, 32.5) | 1.36 (1.06, 1.74) |  | 1.26 (0.95, 1.68) |  |
|  | high |  | 126/137 | 11.7 (10.3, 14.9) | 0.95 (0.74, 1.21) |  | 0.96 (0.74, 1.23) |  |  | 112/137 | 34.0 (28.0, 38.1) | 1.00 (0.77, 1.29) |  | 0.98 (0.75, 1.28) |  |
| GABRQ (scale: 1.68) | continuous |  | 405/433 | 11.1 (10.1, 12.1) | 0.91 (0.82, 1) | 0.054 | 0.92 (0.83, 1.02) | 0.12 |  | 360/433 | 30.6 (27.7, 34.1) | 0.96 (0.86, 1.06) | 0.4 | 1 (0.89, 1.11) | 0.93 |
| GABRQ | low |  | 135/145 | 11.0 (9.3, 12.4) | Reference | 0.45 | Reference | 0.79 |  | 119/145 | 29.4 (25.0, 35.4) | Reference | 0.83 | Reference | 0.47 |
|  | mid |  | 138/148 | 10.9 (9.5, 12.9) | 0.91 (0.72, 1.16) |  | 0.98 (0.77, 1.25) |  |  | 124/148 | 32.5 (26.3, 35.7) | 1.03 (0.80, 1.32) |  | 1.18 (0.91, 1.52) |  |
|  | high |  | 132/140 | 12.7 (9.8, 15.9) | 0.86 (0.67, 1.09) |  | 0.92 (0.72, 1.18) |  |  | 117/140 | 30.6 (26.1, 35.9) | 0.95 (0.74, 1.23) |  | 1.08 (0.83, 1.41) |  |
| GABRR1 (scale: 2.67) | continuous |  | 405/433 | 11.1 (10.1, 12.1) | 0.87 (0.79, 0.97) | **0.0094** | 0.92 (0.82, 1.02) | 0.11 |  | 360/433 | 30.6 (27.7, 34.1) | 0.86 (0.77, 0.96) | **0.007** | 0.92 (0.82, 1.03) | 0.14 |
| GABRR1 | low |  | 140/145 | 10.0 (9.0, 11.3) | Reference | **0.03** | Reference | **0.042** |  | 130/145 | 25.5 (22.5, 29.9) | Reference | **0.0028** | Reference | **0.017** |
|  | mid |  | 131/144 | 11.7 (9.8, 13.5) | 0.75 (0.59, 0.95) |  | 0.72 (0.56, 0.93) |  |  | 115/144 | 33.8 (26.9, 39.4) | 0.68 (0.53, 0.88) |  | 0.69 (0.53, 0.89) |  |
|  | high |  | 134/144 | 12.1 (11.0, 14.7) | 0.77 (0.61, 0.98) |  | 0.85 (0.66, 1.09) |  |  | 115/144 | 34.3 (29.4, 38.7) | 0.69 (0.54, 0.89) |  | 0.79 (0.61, 1.03) |  |
| GABRR2 (scale: 0.86) | continuous |  | 405/433 | 11.1 (10.1, 12.1) | 0.95 (0.86, 1.05) | 0.29 | 0.9 (0.82, 1) | **0.047** |  | 360/433 | 30.6 (27.7, 34.1) | 0.97 (0.87, 1.07) | 0.53 | 0.91 (0.82, 1.01) | 0.089 |
| GABRR2 | low |  | 138/145 | 11.2 (10.1, 13.1) | Reference | 0.72 | Reference | 0.57 |  | 121/145 | 29.2 (25.2, 34.9) | Reference | 0.85 | Reference | 0.48 |
|  | mid |  | 137/146 | 12.0 (10.9, 14.1) | 0.93 (0.74, 1.18) |  | 0.91 (0.71, 1.16) |  |  | 119/146 | 33.4 (28.9, 35.9) | 0.95 (0.74, 1.23) |  | 0.91 (0.70, 1.18) |  |
|  | high |  | 130/142 | 10.0 (8.7, 11.5) | 1.03 (0.81, 1.31) |  | 0.87 (0.67, 1.13) |  |  | 120/142 | 28.2 (25.0, 35.8) | 1.02 (0.80, 1.32) |  | 0.85 (0.65, 1.11) |  |
| ABAT (scale: 1.06) | continuous |  | 405/433 | 11.1 (10.1, 12.1) | 0.83 (0.75, 0.92) | **2.2e-04** | 0.9 (0.8, 1.01) | 0.072 |  | 360/433 | 30.6 (27.7, 34.1) | 0.76 (0.68, 0.84) | **2.6e-07** | 0.81 (0.72, 0.91) | **6e-04** |
| ABAT | low |  | 132/145 | 9.0 (7.5, 11.0) | Reference | **0.0082** | Reference | 0.51 |  | 121/145 | 20.9 (15.6, 26.3) | Reference | **7.1e-05** | Reference | **0.015** |
|  | mid |  | 137/145 | 11.3 (10.1, 13.1) | 0.87 (0.69, 1.11) |  | 0.92 (0.71, 1.18) |  |  | 127/145 | 30.2 (25.5, 34.7) | 0.92 (0.71, 1.18) |  | 0.98 (0.75, 1.27) |  |
|  | high |  | 136/143 | 13.1 (11.1, 17.3) | 0.69 (0.54, 0.88) |  | 0.85 (0.65, 1.12) |  |  | 112/143 | 39.6 (34.4, 45.4) | 0.59 (0.45, 0.76) |  | 0.69 (0.52, 0.92) |  |
| ALDH5A1 (scale: 0.71) | continuous |  | 405/433 | 11.1 (10.1, 12.1) | 0.79 (0.72, 0.87) | **5.5e-06** | 0.85 (0.76, 0.95) | **0.0044** |  | 360/433 | 30.6 (27.7, 34.1) | 0.79 (0.72, 0.87) | **6.7e-06** | 0.87 (0.78, 0.97) | **0.017** |
| ALDH5A1 | low |  | 143/146 | 9.2 (8.4, 10.9) | Reference | **3.6e-06** | Reference | **0.0019** |  | 132/146 | 23.6 (19.6, 26.1) | Reference | **5.3e-06** | Reference | **0.0094** |
|  | mid |  | 131/143 | 12.7 (10.9, 14.4) | 0.65 (0.52, 0.83) |  | 0.70 (0.54, 0.91) |  |  | 116/143 | 32.4 (26.9, 35.7) | 0.64 (0.50, 0.83) |  | 0.71 (0.54, 0.93) |  |
|  | high |  | 131/144 | 12.9 (11.0, 15.9) | 0.55 (0.44, 0.71) |  | 0.62 (0.47, 0.81) |  |  | 112/144 | 39.2 (34.0, 44.7) | 0.55 (0.42, 0.70) |  | 0.66 (0.49, 0.88) |  |
| GAD1 (scale: 2.4) | continuous |  | 405/433 | 11.1 (10.1, 12.1) | 1.28 (1.15, 1.42) | **2.7e-06** | 1.26 (1.13, 1.41) | **3.2e-05** |  | 360/433 | 30.6 (27.7, 34.1) | 1.3 (1.17, 1.45) | **1.2e-06** | 1.27 (1.13, 1.43) | **5.9e-05** |
| GAD1 | low |  | 133/145 | 14.0 (11.9, 16.3) | Reference | **0.00083** | Reference | **0.0025** |  | 115/145 | 40.3 (34.4, 48.2) | Reference | **5.8e-05** | Reference | **0.0023** |
|  | mid |  | 136/144 | 10.9 (9.3, 12.1) | 1.27 (1.00, 1.62) |  | 1.24 (0.96, 1.60) |  |  | 119/144 | 29.4 (25.6, 35.1) | 1.38 (1.07, 1.79) |  | 1.28 (0.98, 1.68) |  |
|  | high |  | 136/144 | 9.3 (7.6, 11.0) | 1.58 (1.24, 2.01) |  | 1.59 (1.22, 2.06) |  |  | 126/144 | 23.5 (16.9, 29.2) | 1.76 (1.37, 2.27) |  | 1.64 (1.24, 2.17) |  |
| GAD2 (scale: 1.4) | continuous |  | 405/433 | 11.1 (10.1, 12.1) | 0.98 (0.89, 1.09) | 0.75 | 0.94 (0.84, 1.05) | 0.26 |  | 360/433 | 30.6 (27.7, 34.1) | 0.99 (0.89, 1.11) | 0.88 | 0.95 (0.84, 1.08) | 0.42 |
| GAD2 | low |  | 147/160 | 11.0 (10.0, 13.3) | Reference | 0.51 | Reference | 0.67 |  | 134/160 | 31.2 (28.2, 35.9) | Reference | 0.97 | Reference | 0.47 |
|  | mid |  | 124/132 | 11.3 (9.2, 13.1) | 1.14 (0.90, 1.45) |  | 1.12 (0.86, 1.45) |  |  | 108/132 | 29.0 (24.8, 35.7) | 1.03 (0.80, 1.33) |  | 1.03 (0.78, 1.36) |  |
|  | high |  | 134/141 | 11.2 (9.5, 12.7) | 1.11 (0.88, 1.41) |  | 1.03 (0.77, 1.36) |  |  | 118/141 | 32.4 (25.6, 35.8) | 1.00 (0.78, 1.28) |  | 0.87 (0.64, 1.18) |  |
| SLC6A1 (scale: 0.86) | continuous |  | 405/433 | 11.1 (10.1, 12.1) | 1.04 (0.94, 1.16) | 0.4 | 1.01 (0.91, 1.13) | 0.86 |  | 360/433 | 30.6 (27.7, 34.1) | 1.08 (0.97, 1.21) | 0.15 | 1.04 (0.92, 1.17) | 0.54 |
| SLC6A1 | low |  | 134/146 | 10.9 (9.2, 13.0) | Reference | **0.0089** | Reference | 0.1 |  | 120/146 | 31.2 (26.7, 35.7) | Reference | **0.012** | Reference | 0.29 |
|  | mid |  | 130/143 | 12.5 (11.1, 15.5) | 0.81 (0.63, 1.03) |  | 0.84 (0.66, 1.08) |  |  | 111/143 | 31.8 (26.4, 40.2) | 0.88 (0.68, 1.13) |  | 0.97 (0.75, 1.27) |  |
|  | high |  | 141/144 | 10.1 (9.0, 11.5) | 1.17 (0.93, 1.49) |  | 1.10 (0.85, 1.41) |  |  | 129/144 | 28.9 (24.9, 34.4) | 1.28 (1.00, 1.64) |  | 1.19 (0.91, 1.55) |  |
| SLC6A13 (scale: 1.64) | continuous |  | 405/433 | 11.1 (10.1, 12.1) | 1 (0.91, 1.11) | 0.97 | 0.95 (0.85, 1.06) | 0.36 |  | 360/433 | 30.6 (27.7, 34.1) | 1.03 (0.93, 1.14) | 0.59 | 0.96 (0.86, 1.08) | 0.51 |
| SLC6A13 | low |  | 134/145 | 11.4 (10.3, 13.1) | Reference | 0.69 | Reference | 0.43 |  | 116/145 | 34.7 (29.9, 40.8) | Reference | **0.028** | Reference | **0.046** |
|  | mid |  | 137/144 | 10.3 (9.2, 12.3) | 1.10 (0.87, 1.40) |  | 1.08 (0.84, 1.38) |  |  | 129/144 | 26.0 (23.1, 32.6) | 1.40 (1.09, 1.81) |  | 1.37 (1.05, 1.78) |  |
|  | high |  | 134/144 | 11.2 (9.2, 14.8) | 1.02 (0.80, 1.29) |  | 0.91 (0.70, 1.18) |  |  | 115/144 | 30.0 (25.2, 34.9) | 1.16 (0.89, 1.50) |  | 1.03 (0.78, 1.37) |  |
| SLC6A11 (scale: 1.65) | continuous |  | 405/433 | 11.1 (10.1, 12.1) | 1.01 (0.91, 1.12) | 0.85 | 0.96 (0.85, 1.08) | 0.45 |  | 360/433 | 30.6 (27.7, 34.1) | 1.06 (0.94, 1.19) | 0.33 | 1.02 (0.9, 1.16) | 0.77 |
| SLC6A11 | low |  | 137/149 | 10.9 (9.3, 12.9) | Reference | 0.99 | Reference | 0.66 |  | 126/149 | 31.1 (26.1, 35.9) | Reference | 0.99 | Reference | 0.73 |
|  | mid |  | 135/142 | 12.0 (10.9, 14.1) | 0.98 (0.78, 1.25) |  | 0.93 (0.72, 1.20) |  |  | 118/142 | 30.5 (26.4, 37.5) | 0.98 (0.77, 1.27) |  | 0.93 (0.71, 1.22) |  |
|  | high |  | 133/142 | 11.0 (9.2, 12.6) | 1.00 (0.78, 1.26) |  | 0.88 (0.68, 1.16) |  |  | 116/142 | 29.4 (25.0, 35.5) | 0.99 (0.77, 1.27) |  | 0.89 (0.68, 1.18) |  |
| SLC6A12 (scale: 1.32) | continuous |  | 405/433 | 11.1 (10.1, 12.1) | 1.13 (1.02, 1.25) | **0.019** | 1.08 (0.97, 1.21) | 0.13 |  | 360/433 | 30.6 (27.7, 34.1) | 1.13 (1.02, 1.25) | **0.021** | 1.1 (0.99, 1.23) | 0.084 |
| SLC6A12 | low |  | 139/146 | 11.5 (10.9, 13.1) | Reference | **0.018** | Reference | 0.14 |  | 121/146 | 34.1 (30.5, 36.9) | Reference | **0.047** | Reference | 0.13 |
|  | mid |  | 132/143 | 14.0 (11.5, 15.9) | 0.95 (0.75, 1.20) |  | 0.96 (0.75, 1.23) |  |  | 116/143 | 33.7 (27.7, 38.6) | 1.08 (0.83, 1.39) |  | 1.11 (0.85, 1.44) |  |
|  | high |  | 134/144 | 9.2 (8.0, 10.3) | 1.31 (1.03, 1.66) |  | 1.23 (0.96, 1.57) |  |  | 123/144 | 24.7 (19.6, 30.0) | 1.35 (1.05, 1.74) |  | 1.31 (1.01, 1.71) |  |
| RELN (scale: 1.16) | continuous |  | 405/433 | 11.1 (10.1, 12.1) | 1.04 (0.93, 1.16) | 0.53 | 0.98 (0.87, 1.1) | 0.75 |  | 360/433 | 30.6 (27.7, 34.1) | 1.09 (0.97, 1.23) | 0.14 | 1.02 (0.9, 1.15) | 0.81 |
| RELN | low |  | 138/145 | 11.2 (9.6, 13.4) | Reference | 0.99 | Reference | 0.71 |  | 115/145 | 30.0 (25.0, 34.9) | Reference | 0.46 | Reference | 0.79 |
|  | mid |  | 139/145 | 12.0 (10.9, 14.2) | 0.99 (0.79, 1.26) |  | 0.95 (0.75, 1.22) |  |  | 123/145 | 33.3 (28.0, 37.1) | 0.97 (0.75, 1.26) |  | 0.92 (0.71, 1.19) |  |
|  | high |  | 128/143 | 10.1 (9.0, 11.4) | 1.01 (0.79, 1.29) |  | 0.89 (0.68, 1.17) |  |  | 122/143 | 29.4 (25.0, 35.1) | 1.13 (0.88, 1.46) |  | 0.98 (0.74, 1.31) |  |
| LRP8 (scale: 0.78) | continuous |  | 405/433 | 11.1 (10.1, 12.1) | 1 (0.9, 1.12) | 0.97 | 1.06 (0.94, 1.2) | 0.36 |  | 360/433 | 30.6 (27.7, 34.1) | 1 (0.89, 1.13) | 0.94 | 1.09 (0.95, 1.24) | 0.22 |
| LRP8 | low |  | 139/146 | 11.0 (9.5, 13.0) | Reference | 0.65 | Reference | 0.52 |  | 127/146 | 32.6 (28.2, 36.1) | Reference | 0.52 | Reference | 0.13 |
|  | mid |  | 138/145 | 11.2 (10.0, 13.0) | 1.07 (0.85, 1.36) |  | 1.16 (0.90, 1.49) |  |  | 115/145 | 31.1 (26.4, 37.1) | 0.92 (0.72, 1.19) |  | 1.01 (0.77, 1.33) |  |
|  | high |  | 128/142 | 11.0 (9.5, 13.3) | 0.96 (0.75, 1.22) |  | 1.06 (0.81, 1.39) |  |  | 118/142 | 26.7 (24.3, 34.4) | 1.07 (0.83, 1.37) |  | 1.30 (0.98, 1.74) |  |
| DAB1 (scale: 0.57) | continuous |  | 405/433 | 11.1 (10.1, 12.1) | 0.91 (0.83, 1.01) | 0.068 | 0.93 (0.84, 1.03) | 0.16 |  | 360/433 | 30.6 (27.7, 34.1) | 0.91 (0.82, 1.01) | 0.085 | 0.93 (0.83, 1.03) | 0.18 |
| DAB1 | low |  | 139/146 | 10.9 (9.1, 11.7) | Reference | 0.12 | Reference | 0.27 |  | 125/146 | 26.9 (23.7, 32.5) | Reference | 0.099 | Reference | 0.17 |
|  | mid |  | 136/144 | 10.9 (9.3, 12.8) | 0.93 (0.73, 1.17) |  | 0.94 (0.74, 1.20) |  |  | 122/144 | 29.9 (25.2, 36.8) | 0.93 (0.72, 1.19) |  | 0.98 (0.76, 1.27) |  |
|  | high |  | 130/143 | 12.6 (11.0, 15.5) | 0.78 (0.62, 0.99) |  | 0.81 (0.62, 1.05) |  |  | 113/143 | 34.4 (30.6, 38.6) | 0.76 (0.59, 0.98) |  | 0.79 (0.60, 1.04) |  |
| DAB2 (scale: 0.67) | continuous |  | 405/433 | 11.1 (10.1, 12.1) | 0.93 (0.84, 1.03) | 0.15 | 0.87 (0.77, 0.98) | **0.022** |  | 360/433 | 30.6 (27.7, 34.1) | 0.98 (0.88, 1.09) | 0.72 | 0.9 (0.79, 1.03) | 0.12 |
| DAB2 | low |  | 132/145 | 11.0 (9.0, 13.2) | Reference | 0.53 | Reference | **0.026** |  | 113/145 | 28.9 (24.7, 34.4) | Reference | 0.74 | Reference | 0.64 |
|  | mid |  | 139/146 | 11.0 (9.2, 12.9) | 1.08 (0.85, 1.38) |  | 1.11 (0.86, 1.45) |  |  | 123/146 | 31.3 (26.9, 37.4) | 1.01 (0.78, 1.31) |  | 0.97 (0.74, 1.29) |  |
|  | high |  | 134/142 | 11.4 (10.3, 13.0) | 0.95 (0.75, 1.21) |  | 0.78 (0.58, 1.05) |  |  | 124/142 | 30.9 (25.6, 35.8) | 1.10 (0.85, 1.42) |  | 0.87 (0.64, 1.19) |  |
| ^*^*P* value was based on log-rank test for PFS and OS in the univariable categorical analysis, and likelihood ratio test in the univariable continuous and multivariable Cox proportional hazards regression model adjusting for age, sex, ECOG performance status, tumor location, number of metastatic sites, KRAS, MSI status, treatment, chemotherapy, PC1, PC2, and PC3. | | | | | | | | | | | | | | | |

**Table S3.** Mutational status of patient-derived CRC organoids.

UA

| **Symbol** | **Protein Change** | **Variant Class** | **Genomic Change** |
| --- | --- | --- | --- |
| TP53 | p.Arg273Cys | SNV | 17:g.7577121G>A |
| ALK | p.Val793Phe | SNV | 2:g.29456541C>A |
| FAT1 | p.Val4559Ile | SNV | 4:g.187509838C>T |
| FAT1 | p.Gly3483Arg | SNV | 4:g.187525632C>T |
| TET2 | p.Gly1060Arg | SNV | 4:g.106158277G>A |
| PTPRT | p.Arg1117His | SNV | 20:g.40735466C>T |
| FANCA | p.Tyr1341Cys | SNV | 16:g.89805686T>C |
| PARP1 | p.Arg330Gln | SNV | 1:g.226573227C>T |
| MED12 | p.Gln2120His | SNV | X:g.70361172G>C |
| SPTA1 | p.Arg1135Trp | SNV | 1:g.158621231T>A |

UK

| **Symbol** | **Protein Change** | **Variant Class** | **Genomic Change** |
| --- | --- | --- | --- |
| TP53 | p.Tyr163His | SNV | 17:g.7578443A>G |
| ALK | p.Pro1262Thr | SNV | 2:g.29432704G>T |
| EPHA5 | p.Asp269Tyr | SNV | 4:g.66467464C>A |
| DNMT1 | p.Met734CysfsTer59 | deletion | 19:g.10262138AT>A |
| MCL1 | p.Glu171del | deletion | 1:g.150551491GTCC>G |
| CTLA4 | p.Pro154Ser | SNV | 2:g.204736103C>T |
| ANKRD26 | p.Gln20del | deletion | 10:g.27389194CTCT>C |
| CD3EAP | p.Arg292Lys | SNV | 19:g.45912101G>A |
| NF1 | F945fs |  |  |

UP

| **Symbol** | **Protein Change** | **Variant Class** | **Genomic Change** |
| --- | --- | --- | --- |
| TP53 | p.Arg248Trp | SNV | 17:g.7577539G>A |
| ASXL1 | p.Gln372Glu | SNV | 20:g.31021115C>G |
| RUNX1 | p.Arg232Trp | SNV | 21:g.36206818G>A |
| FOXP1 | p.Val419Ile | SNV | 3:g.71027078C>T |
| SH2B3 | p.Ala37Val | SNV | 12:g.111856059C>T |
| SOCS1 | p.Ala35Val | SNV | 16:g.11349232G>A |
| CIC |  | SNV | 19:g.42796450A>G |
| CCND3 | p.Ser259Ala | substitution | 6:g.41903782AG>CA |
| MDM2 | p.Ser4Arg | SNV | 12:g.69202269C>A |
| DNMT3B | p.Asp722Glu | SNV | 20:g.31390211T>A |
| PTPRT | p.Arg1157His | SNV | 20:g.40733279C>T |
| MCL1 | p.Glu171del | deletion | 1:g.150551491GTCC>G |
| PAX7 | p.Arg269His | SNV | 1:g.19027166G>A |
| PIK3C2B | p.Glu1463Ter | insertion | 1:g.204399064G>GA |
| VTCN1 | p.Asp230Asn | SNV | 1:g.117695749C>T |
| RFWD2 | p.Asp430His | SNV | 1:g.176015450C>G |
| MAP3K4 | p.Asn160MetfsTer8 | deletion | 6:g.161469775TA>T |
| RPS6KA4 | p.Gln432His | SNV | 11:g.64136035G>C |

US

| **Symbol** | **Protein Change** | **Variant Class** | **Genomic Change** |
| --- | --- | --- | --- |
| KRAS | p.Gly12Ala | SNV | 12:g.25398284C>G |
| TP53 | p.Gly245Ser | SNV | 17:g.7577548C>T |
| SMAD4 | p.Arg361Cys | SNV | 18:g.48591918C>T |
| FLT3 | p.Arg773GlyfsTer8 | deletion | 13:g.28597587CT>C |
| ERBB4 | p.Arg103Cys | SNV | 2:g.212812269G>A |
| EP300 | p.Leu1160Phe | SNV | 22:g.41553389C>T |
| SH2B3 | p.Ser213Arg | SNV | 12:g.111856588C>A |
| CIC | p.Arg415Trp | SNV | 19:g.42793441C>T |
| ZFHX3 | p.Gln3380del | deletion | 16:g.72822033CGCT>C |
| KAT6A | p.Ser396Asn | SNV | 8:g.41834702C>T |
| MCL1 | p.Val76Ile | SNV | 1:g.150551781C>T |
| PTPRD | p.Gln67His | SNV | 9:g.8636708C>G |
| GATA6 | p.Pro144Arg | SNV | 18:g.19751536C>G |
| STAG1 | p.Glu433Ter | SNV | 3:g.136183739C>A |
| INPP4A | p.Lys412Arg | SNV | 2:g.99169305A>G |

V8

| **Symbol** | **Protein Change** | **Variant Class** | **Genomic Change** |
| --- | --- | --- | --- |
| KRAS | p.Gly12Asp | SNV | 12:g.25398284C>T |
| TP53 | p.Arg248Gln | SNV | 17:g.7577538C>T |
| HRAS | p.Gly75Arg | SNV | 11:g.533833C>T |
| SETBP1 | p.Arg143His | SNV | 18:g.42281739G>A |
| APC | p.Arg1114Ter | SNV | 5:g.112174631C>T |
| APC | p.Glu1309AspfsTer4 | deletion | 5:g.112175211TAAAAG>T |
| FGFR4 | p.Val548Met | SNV | 5:g.176522545G>A |
| SOX9 | p.Gln376Ter | SNV | 17:g.70120124C>T |
| DNMT3B | p.Thr353Ala | SNV | 20:g.31380567A>G |
| TCF7L2 | p.Arg448Gln | SNV | 10:g.114920402G>A |
| MED12 | p.Ser1600Arg | SNV | X:g.70354633A>C |
| GPR124 | p.Arg514His | SNV | 8:g.37691579G>A |
| C11orf30 | p.Gln1158Pro | SNV | 11:g.76257040A>C |
| ANKRD11 | p.Gly464Asp | SNV | 16:g.89351559C>T |

UO

| **Symbol** | **Protein Change** | **Variant Class** | **Genomic Change** |
| --- | --- | --- | --- |
| CTNNB1 | p.Ser45Phe | SNV | 3:g.41266137C>T |
| ATM |  | deletion | 11:g.108121422TTTTA>T |
| ATM | p.Glu2621Gly | SNV | 11:g.108203562A>G |
| RET | p.Ser936Pro | SNV | 10:g.43619123T>C |
| PTEN | p.Lys267ArgfsTer9 | deletion | 10:g.89717769TA>T |
| MSH2 | p.Tyr121His | SNV | 2:g.47635689T>C |
| BCR | p.Asp193Asn | SNV | 22:g.23523724G>A |
| BCR | p.Arg1064ValfsTer45 | deletion | 22:g.23653883GGA>G |
| BCR | p.Met1119Thr | SNV | 22:g.23655107T>C |
| DNMT3A | p.Arg597Trp | SNV | 2:g.25467086G>A |
| DNMT3A | p.Pro32Leu | SNV | 2:g.25523090G>A |
| ALK | p.Val402Leu | SNV | 2:g.29606676C>A |
| SUFU | p.Val194Ter | deletion | 10:g.104352460TG>T |
| JAK2 | p.Trp95Arg | SNV | 9:g.5029839T>C |
| JAK2 | p.Ala660Val | SNV | 9:g.5077567C>T |
| AKT1 | p.Glu375Lys | SNV | 14:g.105239264C>T |
| SMO | p.Pro648GlnfsTer128 | deletion | 7:g.128851866GC>G |
| FGFR1 | p.Ser57Pro | SNV | 8:g.38314895A>G |
| FGFR2 | p.Tyr207Cys | SNV | 10:g.123310808T>C |
| ERBB4 | p.Pro1153Ala | SNV | 2:g.212251602G>C |
| ERBB4 | p.Ile658Val | SNV | 2:g.212495294T>C |
| NOTCH1 | p.Pro1443AlafsTer36 | insertion | 9:g.139400022C>CG |
| CREBBP | p.Pro1983GlnfsTer16 | deletion | 16:g.3779099TG>T |
| CASP8 | p.Val171Glu | SNV | 2:g.202136268T>A |
| EZH2 | p.Leu71Met | SNV | 7:g.148543597G>T |
| MTOR | p.Val322TyrfsTer44 | deletion | 1:g.11308027AC>A |
| CD274 | p.Val253Ala | SNV | 9:g.5465574T>C |
| PDCD1 | p.Thr36ProfsTer9 | deletion | 2:g.242795103TG>T |
| EP300 | p.Thr1838Ile | SNV | 22:g.41573228C>T |
| CARD11 | p.Tyr609Cys | SNV | 7:g.2963981T>C |
| CARD11 | p.Arg555GlyfsTer45 | deletion | 7:g.2968322CG>C |
| ASXL1 | p.Pro1322Leu | SNV | 20:g.31024480C>T |
| CUX1 | p.Pro1135Leu | SNV | 7:g.101870887C>T |
| FOXP1 | p.Arg52GlyfsTer32 | deletion | 3:g.71179680CG>C |
| BCL6 | p.Pro191Gln | SNV | 3:g.187447621G>T |
| AKT3 | p.Val242Met | SNV | 1:g.243736323C>T |
| LRP1B | p.Cys1013Tyr | SNV | 2:g.141707902C>T |
| ZFHX3 | p.Leu1269Arg | SNV | 16:g.72845534A>C |
| ZFHX3 | p.Ala714Thr | SNV | 16:g.72991905C>T |
| ERG | p.Asp47Asn | SNV | 21:g.39817445C>T |
| FLI1 | p.Glu108Ter | SNV | 11:g.128638104G>T |
| ACVR1B | p.Asp376Gly | SNV | 12:g.52379000A>G |
| ACVR1B | p.Arg420Ter | SNV | 12:g.52379131C>T |
| KDM6A | p.Tyr119His | SNV | X:g.44833931T>C |
| TCF3 | p.Val357TrpfsTer37 | deletion | 19:g.1620991CG>C |
| KAT6A | p.Pro1713Thr | SNV | 8:g.41790601G>T |
| E2F3 | p.Ala52_Ala53del | deletion | 6:g.20402613TGCCGCC>T |
| PTPRD | p.Arg540His | SNV | 9:g.8507359C>T |
| BMPR1A | p.Pro481Ser | SNV | 10:g.88683231C>T |
| NSD1 | p.Ala1670Asp | SNV | 5:g.176687032C>A |
| NSD1 | p.Ser1937ProfsTer32 | deletion | 5:g.176707748CT>C |
| FLT4 | p.Arg3Trp | SNV | 5:g.180076539G>A |
| TCF7L2 | p.Tyr100Ter | SNV | 10:g.114711285T>G |
| B2M | p.Leu15PhefsTer41 | deletion | 15:g.45003780ACT>A |
| B2M | p.Val69TrpfsTer34 | deletion | 15:g.45007752GA>G |
| TP63 | p.Ala187Thr | SNV | 3:g.189526295G>A |
| PIK3CD | p.Ala849Thr | SNV | 1:g.9783301G>A |
| TSHR | p.Arg310Leu | SNV | 14:g.81609331G>T |
| GRIN2A | p.Phe576Ser | SNV | 16:g.9928012A>G |
| RANBP2 | p.Lys2576AsnfsTer63 | deletion | 2:g.109384717TA>T |
| SPTA1 | p.Ala1372Val | SNV | 1:g.158615057G>A |
| SPEN | p.Leu1091Ala | substitution | 1:g.16256006CT>GC |
| GLI1 | p.Gly274AlafsTer6 | deletion | 12:g.57860074TG>T |
| DOT1L | p.Gly793Ser | SNV | 19:g.2216733G>A |
| SMC3 | p.Tyr43MetfsTer69 | deletion | 10:g.112333493CT>C |
| IRF2 | p.Asp51MetfsTer19 | deletion | 4:g.185340659CT>C |
| FAM46C | p.Thr147ArgfsTer8 | deletion | 1:g.118165929AC>A |
| HIST2H3D | p.Thr23Ala | SNV | 1:g.149785170T>C |
| MAP3K4 | p.Thr77Ile | SNV | 6:g.161455368C>T |
| RPS6KA4 | p.Met566Ile | SNV | 11:g.64137266G>A |
| PAK3 | p.Thr196GlnfsTer87 | deletion | X:g.110406147CA>C |

**Table S4.** GSEA of Hallmark gene sets on gene expression comparisons between model systems.

|  | Chip vs Tumor | Organoid vs Tumor | Organoid vs Chip |
| --- | --- | --- | --- |
|  | padj | padj | padj |
| Hm Adipogenesis | NA | NA | 0.264057161 |
| Hm Allograft Rejection | 2.41657E-09 | 1.49464E-09 | 0.64778217 |
| Hm Androgen Resp | 0.001375175 | 1 | 0.000715273 |
| Hm Angiogenesis | 0.000106823 | 1.5145E-08 | 0.31075419 |
| Hm Apical Junction | 3.18594E-06 | 8.96863E-09 | 0.172655051 |
| Hm Apical Surface | 0.30190678 | 0.375251762 | 0.78808522 |
| Hm Apoptosis | 0.183694084 | 2.19838E-06 | 0.000862262 |
| Hm Bile Acid Met | 0.802606449 | 0.555367709 | 0.172655051 |
| Hm Cholesterol Homeostasis | 1.53697E-06 | NA | 0.028933986 |
| Hm Coagulation | 7.48534E-08 | 6.44617E-14 | 0.031040601 |
| Hm Complement | 0.000302455 | 5.5395E-09 | 0.001091401 |
| Hm Dna Repair | 0.627273073 | NA | 0.066059894 |
| Hm E2f Targets | NA | NA | 0.788351191 |
| Hm Epithelial Mesenchymal Transition | 9.72441E-28 | 4.27429E-37 | 0.027690196 |
| Hm Estrogen Resp Early | NA | 1 | 3.47062E-05 |
| Hm Estrogen Resp Late | NA | 1 | 0.000515551 |
| Hm Fatty Acid Met | 5.5836E-07 | NA | 0.00190008 |
| Hm G2m Checkpoint | NA | NA | 0.272785623 |
| Hm Glycolysis | NA | NA | 0.305029109 |
| Hm Hedgehog Sig | 0.280181723 | 0.027814244 | 0.509762111 |
| Hm Heme Met | NA | 1 | 0.000933759 |
| Hm Hypoxia | 0.131282737 | 0.000313828 | 0.166672849 |
| Hm Il2 Stat5 Sig | 0.001375175 | 0.000313828 | 0.376299945 |
| Hm Il6 Jak Stat3 Sig | 0.000171913 | 1.15632E-06 | 0.248015873 |
| Hm Inflammatory Resp | 1.64415E-10 | 7.68929E-14 | 0.000704096 |
| Hm Interferon Alpha Resp | 0.042532166 | 0.000795422 | 0.12292527 |
| Hm Interferon Gamma Resp | 1.69036E-07 | 6.58716E-10 | 0.066059894 |
| Hm Kras Sig Dn | 0.000487285 | 2.88006E-06 | 0.226150725 |
| Hm Kras Sig Up | 2.68685E-07 | 9.53694E-15 | 0.028933986 |
| Hm Mitotic Spindle | NA | NA | 0.826044704 |
| Hm Mtorc1 Sig | NA | NA | 0.001646747 |
| Hm Myc Targets V1 | NA | NA | 0.127876589 |
| Hm Myc Targets V2 | 0.063018851 | NA | 0.361599546 |
| Hm Myogenesis | 2.68685E-07 | 6.54177E-11 | 0.165636593 |
| Hm Notch Sig | 0.618489583 | 0.603612815 | 0.826044704 |
| Hm Oxidative Phosphorylation | NA | NA | 0.028933986 |
| Hm P53 Path | 0.928857715 | 1 | 0.132903901 |
| Hm Pancreas Beta Cells | 0.562102851 | 0.191295547 | 0.165898618 |
| Hm Peroxisome | 0.000527023 | NA | 0.044957521 |
| Hm Pi3k Akt Mtor Sig | 0.005531386 | NA | 0.06058597 |
| Hm Protein Secretion | 5.70711E-11 | NA | 3.47368E-06 |
| Hm Reactive Oxygen Species Path | 0.095244923 | NA | 0.053888021 |
| Hm Spermatogenesis | 0.694141486 | 0.411428571 | 0.994818653 |
| Hm Tgf Beta Sig | 0.78754118 | 0.000313828 | 0.000821472 |
| Hm Tnfa Sig Via Nfkb | 5.47778E-06 | 3.90933E-12 | 3.47368E-06 |
| Hm Unfolded Protein Resp | 3.58882E-16 | NA | 7.12266E-05 |
| Hm Uv Resp Dn | 2.60084E-06 | 1.98788E-11 | 0.002054662 |
| Hm Uv Resp Up | 0.056137357 | 0.342570473 | 0.00413105 |
| Hm Wnt Beta Catenin Sig | 0.825114168 | 0.042727665 | 0.737311385 |
| Hm Xenobiotic Met | NA | 1 | 0.004219087 |
